# Inflammation-Driven Regulation of PD-L1 and PD-L2, and Their Cross-Interactions with Protective Soluble TNFα Receptors in Human Triple-Negative Breast Cancer

**DOI:** 10.3390/cancers14143513

**Published:** 2022-07-19

**Authors:** Tamir Baram, Nino Oren, Nofar Erlichman, Tsipi Meshel, Adit Ben-Baruch

**Affiliations:** The Shmunis School of Biomedicine and Cancer Research, George S. Wise Faculty of Life Sciences, Tel Aviv University, Tel Aviv 6997801, Israel; tamiros21@gmail.com (T.B.); nino.or64@gmail.com (N.O.); nofarerlichman@gmail.com (N.E.); tsipi.meshel@gmail.com (T.M.)

**Keywords:** breast cancer, interferon γ, interleukin 1β, PD-L1/PD-L2, pro-inflammatory cytokines, soluble TNFR1/soluble TNFR2, tumor necrosis factor α

## Abstract

**Simple Summary:**

Immune checkpoint blockades (ICBs) to PD-L1 have led to major breakthroughs in cancer therapy, but in triple-negative breast cancer (TNBC) success rates are rather limited. Following studies suggesting that chronic inflammation may limit ICB efficacy, we found that pro-inflammatory cytokines up-regulated the proportion of TNBC cells co-expressing the inhibitory immune checkpoint PD-L1 and its cognate PD-L2 molecule. Moreover, we demonstrated that in the context of inflammation-driven signals, PD-L1 down-regulated the cell-derived levels of sTNFR1 and sTNFR2, the soluble receptors of tumor necrosis factor α (TNFα); these soluble receptors were found to exert protective/anti-metastatic effects in TNBC cells, manifested by their ability to down-regulate TNFα-induced production of pro-metastatic chemokines by TNBC cells. Our findings possibly testify for a novel mechanism of PD-L1-mediated tumor progression in which PD-L1 prevents the anti-metastatic effects of sTNFR1 and sTNFR2 in TNBC cells. This mechanism may also act in vivo, in parallel to immune suppression under inflammatory conditions.

**Abstract:**

Pro-inflammatory cytokines play key roles in elevating cancer progression in triple-negative breast cancer (TNBC). We demonstrate that specific combinations between TNFα, IL-1β and IFNγ up-regulated the proportion of human TNBC cells co-expressing the inhibitory immune checkpoints PD-L1 and PD-L2: TNFα + IL-1β in MDA-MB-231 cells and IFNγ + IL-1β in BT-549 cells; in the latter cells, the process depended entirely on STAT1 activation, with no involvement of p65 (CRISPR-Cas9 experiments). Highly significant associations between the pro-inflammatory cytokines and PD-L1/PD-L2 expression were revealed in the TCGA dataset of basal-like breast cancer patients. In parallel, we found that the pro-inflammatory cytokines regulated the expression of the soluble receptors of tumor necrosis factor α (TNFα), namely sTNFR1 and sTNFR2; moreover, we revealed that sTNFR1 and sTNFR2 serve as anti-metastatic and protective factors in TNBC, reducing the TNFα-induced production of inflammatory pro-metastatic chemokines (CXCL8, CXCL1, CCL5) by TNBC cells. Importantly, we found that in the context of inflammatory stimulation and also without exposure to pro-inflammatory cytokines, elevated levels of PD-L1 have down-regulated the production of anti-tumor sTNFR1 and sTNFR2. These findings suggest that in addition to its immune-suppressive activities, PD-L1 may promote disease course in TNBC by inhibiting the protective effects of sTNFR1 and sTNFR2.

## 1. Introduction

Immune checkpoint blockades (ICBs) that target the immune inhibitory molecules CTLA-4, PD-1 and PD-L1 have opened a new era in cancer therapy. In addition to melanoma, in which the efficacy of ICBs is clearly evident, these immunotherapies are currently assessed in a large variety of malignancies [1,2]. Between others, ICBs targeting PD-1 and its ligand PD-L1 have been introduced to the treatment of the most aggressive subtype of triple-negative breast cancer (TNBC), corresponding mainly to the basal-like type of breast cancer in genomic TCGA studies [3,4]. TNBC tumors are negative for the expression of estrogen receptors and progesterone receptors and do not over-express HER2, which are the most applicable targets in breast cancer therapy. Hence, TNBC patients are treated systemically by chemotherapy, followed by relapse and recurrence in many of the patients [3,5].

Clinical trials that were performed in TNBC patients demonstrated a relatively limited efficacy of ICBs, which was increased when chemotherapies were administered in parallel. However, even under these favorable conditions, many TNBC patients did not reach complete or even partial response to treatment [6,7,8]. The mechanisms limiting the efficacy of ICBs in cancer therapy have been the subject of intensive research, raising, among others, the possibility that chronic tumor inflammation—that is considered “The Seventh Hallmark of Cancer” [9,10,11,12]—counteracts the beneficial impact of ICBs and reduces their effectiveness [13,14,15].

Within the inflammatory tumor microenvironment (TME), the potent pro-inflammatory cytokines tumor necrosis factor α (TNFα) and interleukin 1β (IL-1β) generally have major tumor- and metastasis-supporting roles; the pro-cancer functions of these two cytokines are diverse, affecting the cancer cells and the TME [11,12,16,17,18,19,20,21,22]. Whereas TNFα and IL-1β are minimally expressed in normal breast tissues, their expression levels in breast cancer patients are relatively high, and are increased as disease progresses [18,20,23,24,25,26,27], and studies in breast cancer animal models provided evidence to their causative roles in promoting disease course [18,19,28,29,30,31,32].

Another important cytokine that is strongly connected to breast cancer progression is interferon γ (IFNγ). IFNγ is an important inducer of acquired immune activities, but under certain conditions it leads to immune shut-off by inducing the expression of PD-L1 [33,34,35]. At the cancer context, IFNγ was found to have anti-malignancy as well as pro-malignancy effects, the latter partly mediated by the ability of this cytokine to promote PD-L1 expression in immune and tumor cells; moreover, under certain conditions, IFNγ can turn into a potent pro-inflammatory factor that contributes to cancer progression [34,35,36,37,38,39,40].

In addition to the IFNγ connection with PD-L1 expression, several publications demonstrated that TNFα and its receptors (TNFR1 and TNFR2) are connected to the PD-L1/PD-1 axis. For example, the addition of TNFα/TNFRs inhibitors to the blockade of PD-L1/PD-1 has potentiated anti-tumor effects in animal cancer models [41,42,43,44]. Furthermore, our studies demonstrated the connection between the TNFα-TNFR2 axis and the PD-L1/PD-1 axis in TNBC patients and in a TNBC mouse model [45,46]. In parallel, investigations indicated that IL-1β blocking has cooperated with anti-PD-1 treatment in reducing tumor growth in a mouse breast cancer model [47] and that IL-1β could up-regulate PD-L1 expression [48,49].

The connections of TNFα, IL-1β and IFNγ with the PD-L1/PD-1 axis, as well as with cancer-related inflammation, have led us to characterize the impact of these cytokines—each alone and in different combinations—on the expression of PD-L1 by TNBC cells. Moreover, we have investigated the impact of the pro-inflammatory cytokines on PD-L2 expression by the cancer cells. Emerging findings have recently demonstrated that PD-L2, the other PD-1 ligand, is expressed by cancer cells and is connected to poor prognosis, including in breast cancer, presumably by its ability to down-regulate immune activities [50,51,52,53,54].

In parallel, in view of the major roles of chronic inflammation in promoting TNBC progression, we studied the soluble forms of TNFR1 and TNFR2, namely sTNFR1 and sTNFR2. sTNFR1 and sTNFR2 can down-regulate inflammatory conditions by competing with membrane TNFR1 (mTNFR1) and mTNFR2 on TNFα binding and activities [55,56,57,58,59], and are detected in breast cancer patients [60,61,62,63,64,65]. In this study, we asked how sTNFR1 and sTNFR2 control the ability of the potent pro-inflammatory cytokine TNFα to induce the expression of inflammatory pro-metastatic chemokines by TNBC cells. Then, the regulation of sTNFR1 and sTNFR2 expression by PD-L1 was explored, under conditions of pro-inflammatory stimulation and in its absence.

The observations of the current study reveal that pro-inflammatory cytokines act in cooperativity to induce the proportion of TNBC cells co-expressing the inhibitory immune checkpoints PD-L1 + PD-L2. Moreover, the pro-inflammatory cytokines control the expression of sTNFR1 and sTNFR2, which act as potential anti-metastatic factors by reducing the release of inflammatory pro-metastatic chemokines by TNFα-stimulated TNBC cells. Then, by studying TNBC cells that express high vs. absent/low levels of PD-L1, we demonstrate that elevated PD-L1 expression down-regulates the levels of the anti-metastatic cell-derived soluble receptors, sTNFR1 and sTNFR2, in the presence and absence of pro-inflammatory stimulation.

Thus, in the context of cancer-related inflammation, the increase in PD-L1 expression by pro-inflammatory cytokines may provide TNBC cells with two advantages: protection against immune surveillance and down-regulation of the production of anti-metastatic factors. By providing these novel findings, our study demonstrates that an important link exists between pro-inflammatory factors, immune checkpoints and sTNFR1/sTNFR2, and sheds light on their potential roles in controlling the malignancy potential of TNBC cells.

## 2. Materials and Methods

### 2.1. Cell Cultures

Human MDA-MB-231 [MDA; American Type Culture Collection (ATCC)] and MDA-MB-468 cells (ATCC) were cultured in growth medium containing DMEM (4.5 g/L glucose) supplemented by 10% fetal bovine serum (FBS), 2% L-glutamine and 1% penicillin-streptomycin-amphotericin solution (all materials are from Biological Industries, Beit Háemek, Israel). BT-549 cells (BT; ATCC) were cultured in growth medium containing RPMI 1640 (4.5 g/L glucose) supplemented by 10% FBS, 1% penicillin-streptomycin (all from Biological Industries) and 0.1% insulin (#I9278; Sigma-Aldrich, Saint Louis, MO, USA).

When indicated, the study included MDA cells and BT cells that were manipulated to over-express PD-L1, as described in [66]. Briefly, in MDA cells we have compared between cells that expressed PD-L1 and cells that did not express PD-L1 at any level. To generate such cells, the endogenous expression of PD-L1 in MDA cells was knocked out (KO) by CRISPR-Cas9. Then, the cells were infected to over-express WT PD-L1 or infected by an empty vector control. In parallel, BT cells that expressed endogenous PD-L1 were infected to over-express WT PD-L1 or infected by an empty vector control. These BT cells differed from those used in our published study [66] by the fact that they did not express mCherry.

### 2.2. Stimulation by Pro-Inflammatory Cytokines

Unless otherwise indicated, the cells were stimulated by recombinant human (rh) TNFα (50 ng/mL; #300-01A, PeproTech, Rocky Hill, NJ, USA), rhIL-1β (500 pg/mL, #200-01B, PeproTech) and/or rhIFNγ (20 ng/mL, #300-02B, PeproTech). The cytokine concentrations were selected based on titration analyses that were performed beforehand, and they agree with the conventional dose range used in other research systems (TNFα: [67,68,69]; IL-1β: [70,71,72]; IFNγ: [73,74]). In all procedures, control non-stimulated cells were treated with the vehicle of the stimulating cytokines (0.1% BSA diluted in double distilled water).

In experiments determining PD-L1 and PD-L2 expression by flow cytometry, and in ELISA studies determining chemokine expression, the cells were exposed to cytokines/vehicle for 24 h; in ELISA studies determining sTNFR1 and sTNFR2 extracellular expression, the cells were stimulated by the cytokines/vehicle for 48–97 h, as indicated in figure legends; in quantitative real-time PCR (qPCR) analyses (TNFR1 and TNFR2 mRNA), duration of stimulation was 5 h, in order to visualize transcription-related effects prior to protein synthesis; in Western blot (WB) experiments studying p65 and STAT1 activation, the cells were treated with the cytokines/vehicle for different time points, as indicated in the figures. 

### 2.3. Flow Cytometry Analyses of PD-L1 and PD-L2 Expression

In single-stain analyses of PD-L1 or PD-L2 expression by MDA cells, unconjugated mouse IgG1 antibodies against human PD-L1 (#14-5983-82, 0.5 µg/test, eBioscience, San Diego, CA, USA) or against human PD-L2 (#14-5888-82, 0.5 µg/test, eBioscience or #345502, 0.5 µg/test, Biolegend, San Diego, CA, USA (same clones)) were used. Then, FITC-conjugated goat anti-mouse IgG (#115-095-003, 1.5μg/test, Jackson ImmunoResearch Laboratories, West Grove, PA, USA) were used as secondary antibodies. In single-stain analyses of PD-L1 expression in BT cells, PE-conjugated mouse IgG1 antibodies against human PD-L1 (#12-5983-42, 0.5 µg/test, eBioscience) were used, and single staining of PD-L2 was performed as in MDA cells.

To determine co-expression of PD-L1 and PD-L2, the cells were first stained by unconjugated mouse IgG1 antibodies against human PD-L2 (#14-5888-82, 0.5 µg/test, eBioscience) followed by FITC-conjugated goat anti-mouse IgG (#115-095-003, 1.5 μg/test, Jackson ImmunoResearch); then, staining was performed with PE-conjugated mouse IgG1 against human PD-L1 (#12-5983-42, 0.5 µg/test, eBioscience).

Cell surface expression of PD-L1 and/or PD-L2 was determined using S1000EXi flow-cytometer (Stratedigm, San Jose, CA, USA). Baseline staining was determined by non-relevant isotype-matched antibodies used as controls (#400102, 0.5 µg/test, or #400114, 0.5 µg/test, Biolegend). Analyses were performed using the “Flow-Jo V10” software (BD Biosciences, Haryana, India).

### 2.4. Analyses of Patient Datasets

The TCGA dataset [75], was used to determine RNAseq-based gene expression values in basal-like patients (*n* = 141). Patients belonging to this subtype were identified by the PAM50 annotation file of the dataset. Correlation coefficients and *p* values were determined by Spearman rank rho. *p* ≤ 0.05 values were considered statistically significant.

### 2.5. Western Blot Analyses

Following cytokine stimulation, cells were lysed in RIPA lysis buffer containing phosphatase and protease inhibitors, followed by conventional WB and transfer procedures. The following primary rabbit IgG antibodies were used (unless otherwise indicated, all antibodies were from Cell Signaling Technology, Danvers, MA, USA): Phosphorylated (P)-p65(p-Ser-536) #3033, 1:1000; Total (T)-p65 #8242, 1:1000; P-STAT1(p-Tyr701) #9167, 1:1000; T-STAT1 #9172, 1:1000. Rabbit IgG antibodies against GAPDH (#ab9485; 1:10,000, Abcam, Cambridge, UK) served as loading controls. Then, the membranes were reacted with streptavidin-horseradish peroxidase (HRP)-conjugated goat anti-rabbit IgG (#111-035-003; 1:10,000, Jackson ImmunoResearch Laboratories). The membranes were subjected to enhanced chemiluminescence (#20-500, Biological Industries), and were visualized using Kodak Medical X-RAY processor (Carestream Health, Rochester, NY, USA) or Amersham Imager 600 (GE Healthcare, Little Chalfont, UK). File S1. Full western blot images can be found in Supplementary Materials.

### 2.6. Generating STAT1 and p65 KO Cells

The CRISPR/Cas9 system was employed in order to KO endogenous STAT1 or p65 in BT cells. Small guided RNA (sgRNA) targeting STAT1 (GAGGTCATGAAAACGGATGG) and sgRNA targeting GFP as control (GGGCGAGGAGCTGTTCACCG), were cloned into pXPR lenti-CRISPR plasmid (puromycin resistance) at the BsmBI site [76]; they were kindly provided by Prof. Bacharach and Prof. Ehrlich (Shmunis School of Biomedicine and Cancer Research, Tel Aviv University). sgRNA targeting p65 (TCCTTTCCTACAAGCTCGTG) (Sigma-Aldrich) was designed using the crispr.mit.edu web tool and cloned into the same plasmid. Lentiviral particles containing the pXPR lentivectors and the above sgRNAs were prepared and used for infection of BT cells. Following selection by growth in 2 µg/mL puromycin dihydrochloride (#1033, AG Scientific, San Diego, CA, USA), appropriate clones were pooled. The lack of STAT1 or p65 expression and activation in pooled KO cells was validated by WB analyses.

### 2.7. ELISA Analyses

Cell conditioned media (CM) were collected from cancer cells, cleared by centrifugation, and used in ELISA assays. The expression of all factors was detected in 24–96-h cultures, as indicated in figure legends. Analyses were performed in parallel to standard proteins and at the linear range of absorbance.

Levels of sTNFR1 and sTNFR2 were determined using designated ELISA kits: Human TNFRI/TNFRSF1A DuoSet (#DY225, R&D Systems, Minneapolis, MN, USA) for sTNFR1 and Human sTNFRII/TNFRSF1B DuoSet (#DY726, R&D Systems) for sTNFR2. In the experiments with PD-L1 over-expressing cells, determination of sTNFR1 and sTNFR2 was performed using the following antibodies and recombinant proteins (all from R&D Systems): sTNFR1: Coating antibodies #MAB625, 4 μg/mL; Detecting antibodies #BAF225, 0.025 μg/mL; sTNFR1 standard protein #636-R1. sTNFR2: Coating antibodies #MAB726, 2 μg/mL; Detecting antibodies #BAF726, 0.15μg/mL; sTNFR2 standard protein #1089-R2.

Chemokine expression levels were determined using the following antibodies (all from PeproTech, unless otherwise indicated): CXCL8: Coating antibodies #500-P28, 0.6 μg/mL; Detecting antibodies #500-P28BT, 0.15 μg/mL; rhCXCL8 standard protein #200-08. CXCL1: Coating antibodies #500-P92, 0.5 μg/mL; Detecting antibodies #500-P92BT, 0.25 μg/mL; rhCXCL1 standard protein #300-11. CCL5: Coating antibodies #500-M75, 7 μg/mL; Detecting antibodies #BAF278, 0.05μg/mL (R&D Systems); rhCCL5 standard protein #300-06. CCL2: Coating antibodies #500-M71, 2.5 μg/mL; Detecting antibodies #500-P34BT, 0.15 μg/mL; rhCCL2 standard protein #300-04.

HRP-conjugated streptavidin (#016-030-084, 0.1 μg/mL, Jackson ImmunoResearch Laboratories) and substrate TMB/E solution (#ES001, Millipore, Burlington, MA, USA) were added, the reaction was stopped by addition of 0.18 M H_2_SO_4_ and absorbance was measured at 450 nm.

When sTNFR1, sTNFR2 and chemokine levels were detected following MMP/ADAM17 inhibition, the cells were incubated with marimastat (1.5μg/mL; #M2699, Sigma-Aldrich) and/or TAPI-0 (5μg/mL; #SML1292, Sigma-Aldrich) for 3 h prior to and during TNFα stimulation. CM were collected, and the soluble factors were determined as indicated above, by ELISA. Inhibitor concentrations were selected based on titration experiments and literature search. In all procedures, control cells were grown in the presence of vehicle of the inhibitors.

### 2.8. Analyses Using Recombinant Soluble TNFR1 and TNFR2

To determine the impact of recombinant soluble (rs) TNFR1 and rsTNFR2 on induction of TNFα-induced chemokine production, rsTNFR1 (150 ng/mL, #636-R1, R&D Systems), rsTNFR2 (500 ng/mL, #1089-R2, R&D Systems), both of them together or a vehicle control were incubated with TNFα (0.5 ng/mL, #300-01A, PeproTech) for 60 min at room temperature. Then, TNFα was added to the cell culture and after 24 h CM were collected and chemokine expression was determined by ELISA. When indicated, 3 h prior and during TNFα stimulation, TAPI-0 was added to the cell culture medium (concentration as above). The concentrations of rsTNFR1 and rsTNFR2 were selected based on titration analyses performed in our lab.

### 2.9. qPCR Analyses

Total RNA was extracted using the EZ-RNA kit (#20-400; Biological Industries) or TRI-Reagent (#T9424, Sigma-Aldrich) and 1-Bromo-3-chloropropane (#B62404, Sigma-Aldrich). Using qScript cDNA Synthesis Kit (#95047, Quantabio, Beverly, MA, USA) containing the MMLV reverse transcriptase and ribonuclease protein, first-strand cDNA was generated from RNA samples. cDNA targets were quantified by CFX Connect Real Time System (Bio-Rad, Hercules, CA, USA) in Hard-Shell PCR 96-well, thin wall plates, (#HSP9601, Bio-Rad). iTaq Universal SYBR Green Supermix (#1725124, Bio-Rad) was used to detect transcripts, according to manufacturer’s instructions. Two pairs of specific primers were used, designed to span different exons. Data were normalized to the housekeeping gene Ribosomal Protein S9 (RS9). Dissociation curves for each primer set indicated a single product, and “no-template” controls were negative after 40 cycles. Quantification was performed by standard curves, within the linear range of quantification. TNFR1 primers: S-GAGGCCATAGCTGTCTGGCA, AS-TTCCCACCAACAGCTCCAGG. TNFR2 primers: S-CCGCCCAGGTGGCATTTACA, AS-TGTCCGAGGTCTTGGTACAGA.

### 2.10. Data Presentation and Statistical Analyses

All experiments were performed in *n* ≥ 3 independent experimental repeats (rarely, significant statistical values in a few of the experiments were not reproducible, but the different experimental repeats of the same experiment have shown the same pattern). Statistical significance was determined by two-tailed unpaired Student’s *t*-tests; in ELISA and qPCR assays, *p* values are presented after adjustment for multiplicity of comparisons, that were carried out using the Benjamini–Hochberg procedure controlling the FDR at 0.05. *p* ≤ 0.05 values were considered statistically significant.

## 3. Results

### 3.1. Pro-Inflammatory Cytokines Act in Cooperativity to Promote the Proportions of TNBC Cells Co-Expressing PD-L1 + PD-L2

In determining the impact of pro-inflammatory cytokines on the expression of PD-L1 and PD-L2 in TNBC cells, we have analyzed the effects of TNFα, IL-1β and IFNγ, each alone and in all possible combinations, in pairs and as a triplet stimulation (cytokine concentrations were selected as described in Section 2). Moreover, in view of the fact that both PD-L1 and PD-L2 were found to be expressed by breast tumor cells in patients and to be associated with each other [50,51,52,53,77], we have also analyzed the proportions of TNBC cells that co-expressed PD-L1 and PD-L2 together.

**Figure 1:**
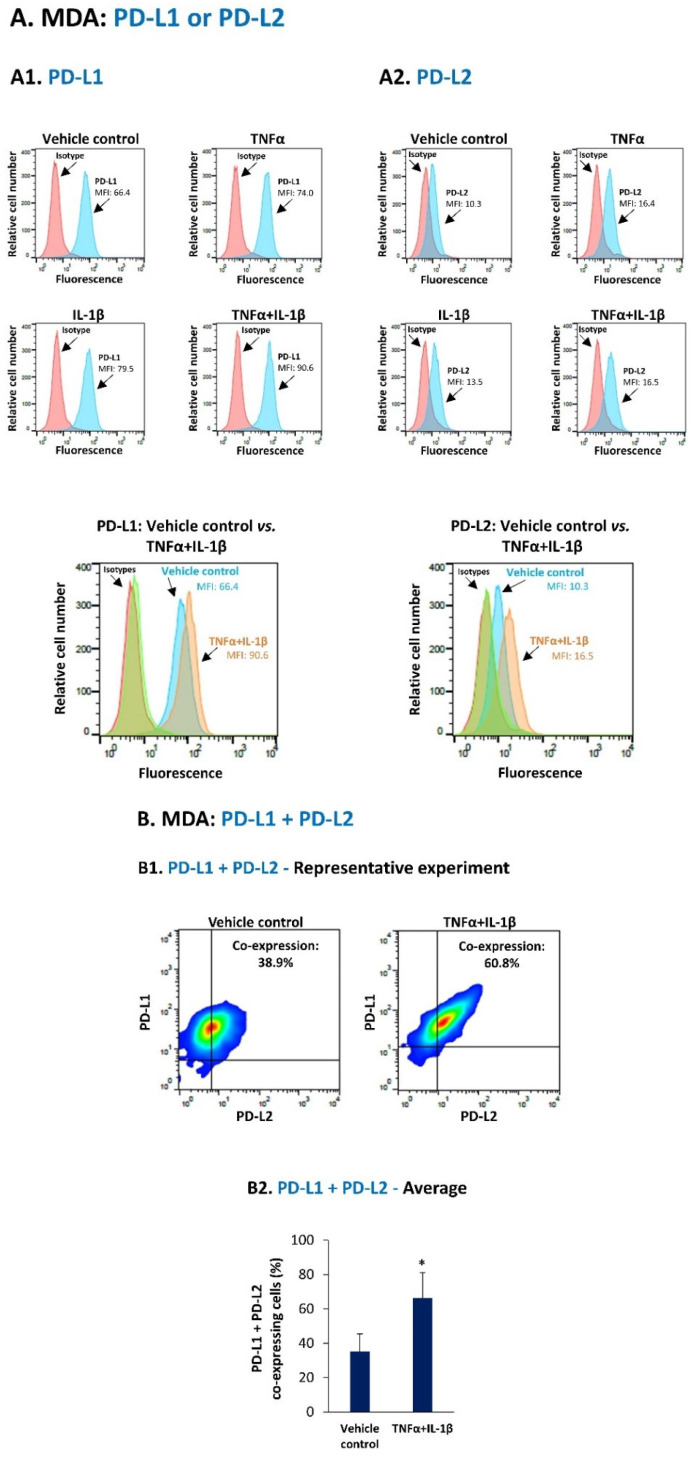
Joint stimulation by TNFα + IL-1β promotes the proportion of PD-L1 + PD-L2 co-expressing MDA-MB-231 cells. (**A**) MDA-MB-231 cells (MDA) were stimulated by TNFα and/or IL-β (TNFα: 50 ng/mL; IL-1β: 500 pg/mL) for 24 h. Control cells were treated by the vehicle of the cytokines. Cytokine concentrations were selected as described in Section 2. Cell surface expression of PD-L1 (**A1**) and PD-L2 (**A2**) was determined by flow cytometry; MFI, mean fluorescence intensity. Isotype/s, Non-relevant isotype-matched antibodies, used as control/s. A representative experiment of *n* = 3 is presented. (**B**) MDA cells were stimulated by TNFα + IL-1β or vehicle, as in Part A. The proportion of cells co-expressing PD-L1 + PD-L2 was determined by flow cytometry. Axes determining PD-L1 + PD-L2 positive cells were set based on isotype staining; The percentages of PD-L1 + PD-L2 co-expressing cells were calculated by subtracting the percentages of PD-L1 + PD-L2 co-expressing cells in isotype-labeled cells from the percentages of PD-L1 + PD-L2 co-expressing cells, following staining by specific antibodies to PD-L1 and PD-L2. (**B1**) A representative experiment of *n* = 3 is presented. (**B2**) Average ± SD of *n* = 3 experiments is presented. * *p* < 0.05. Statistical analyses were performed as described in Section 2.

**Figure 2:**
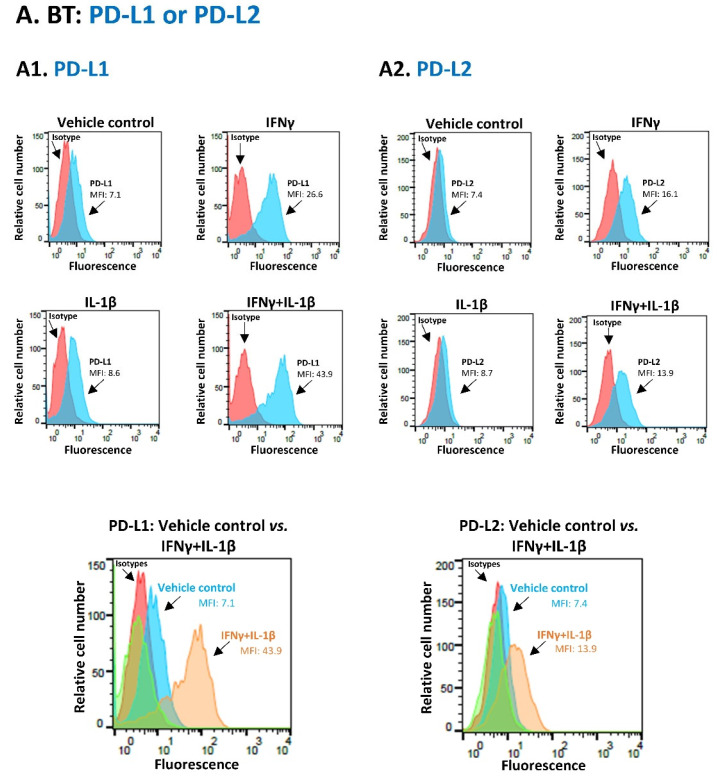

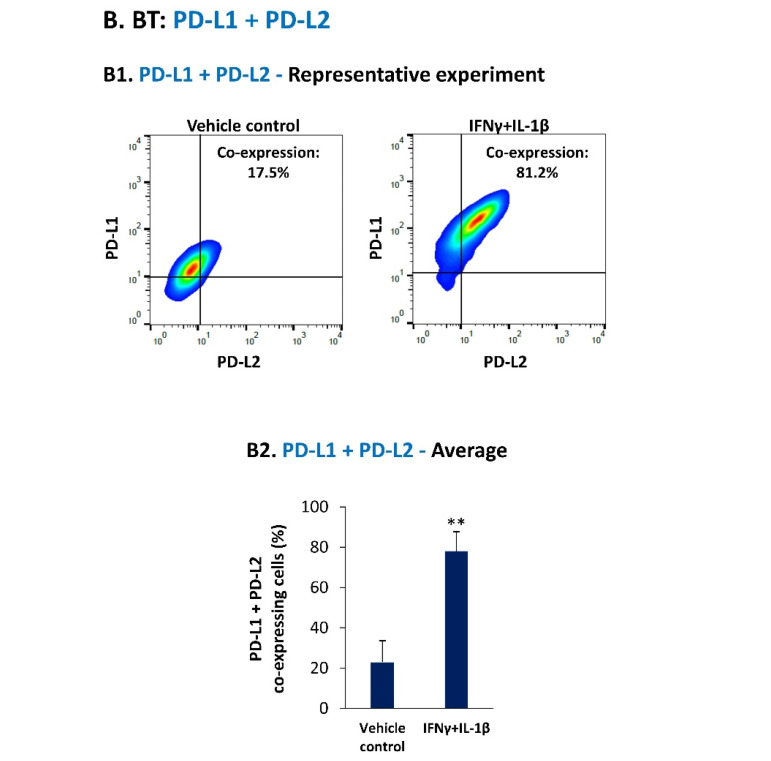
Joint stimulation by IFNγ + IL-1β promotes the proportion of PD-L1 + PD-L2 co-expressing BT-549 cells. (**A**) BT-549 cells (BT) were stimulated by IFNγ and/or IL-β (IFNγ: 20 ng/mL; IL-1β: 500 pg/mL) for 24 h. Control cells were treated by the vehicle of the cytokines. Cytokine concentrations were selected as described in Section 2. Cell surface expression of PD-L1 (**A1**) and PD-L2 (**A2**) was determined by flow cytometry; MFI, mean fluorescence intensity. Isotype/s, Non-relevant antibodies used as control/s. A representative experiment of *n* = 3 is presented. (**B**) BT cells were stimulated by IFNγ + IL-1β or vehicle, as in Part A. The proportion of cells co-expressing PD-L1 + PD-L2 was determined by flow cytometry, as described in Figure 1B. (**B1**) A representative experiment of *n* = 3 is presented. (**B2**) Average ± SD of *n* = 3 experiments is presented. ** *p* < 0.01. Statistical analyses were performed as described in Section 2.

The data of Figure 1 and Figure 2 demonstrate that PD-L1 was constitutively expressed in the human TNBC cells used in our study (vehicle control cells)—MDA-MB-231 (MDA) cells and BT-549 (BT) cells—with MDA cells expressing the protein at higher endogenous levels than BT cells. Then, these figures show the most effective cytokine combinations in promoting the proportion of MDA cells and BT cells co-expressing PD-L1 + PD-L2; all other combinations have led to less prominent effects or did not induce any change in this parameter.

Specifically, in MDA cells, the combined stimulation by TNFα and IL-1β promoted the expression levels of PD-L1, more than did each cytokine alone (determined by MFI (mean fluorescence intensity)) (Figure 1(A1); this is clearly shown in the combined histogram, demonstrating elevated PD-L1 levels in TNFα + IL-1β-stimulated cells compared to vehicle-control cells (by 1.47 ± 0.09 folds, *p* = 0.01, *n* = 3). The combination of these two cytokines also up-regulated the expression levels of PD-L2 (by 1.57 ± 0.24 folds, *p* = 0.05, *n* = 3) (Figure 1(A2); MFI). Following TNFα + IL-1β stimulation, a most prominent elevation was noted in the percentage of cells that co-expressed PD-L1 + PD-L2, simultaneously (Figure 1B).

Similar findings were observed in BT cells, where the most prominent induction of PD-L1 expression levels (MFI) took place when IFNγ and IL-1β were used together, higher than the induction seen with each cytokine alone (Figure 2(A1)). The combined stimulation by IFNγ + IL-1β also promoted PD-L2 expression levels by BT cells (Figure 2(A2)) and led to a pronounced increase in the percentage of cells that co-expressed PD-L1 + PD-L2 together (Figure 2B). The cytokine combination that affected MDA cells—namely TNFα + IL-1β—had a less noticeable effect on the proportion of BT cells co-expressing PD-L1 + PD-L2 than the IFNγ + IL-1β combination (Figure S1 vs. Figure 2B).

**Figure 3:**
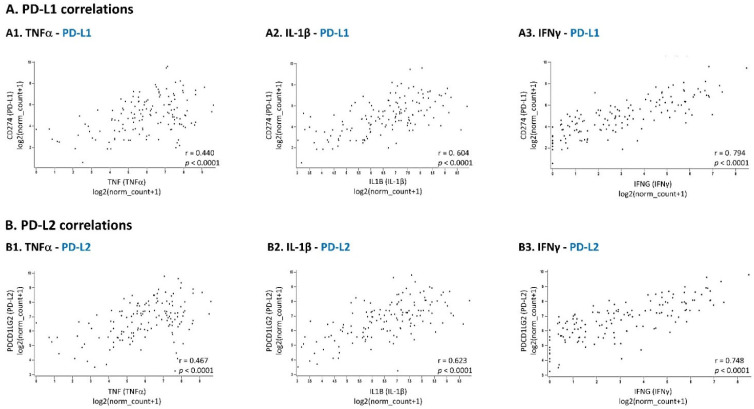
TCGA dataset analyses of basal-like breast cancer patients reveal a highly significant correlation between the expression of TNFα, IL-1β and IFNγ and the expression of PD-L1 and PD-L2. The TCGA breast cancer dataset was used to identify correlations in gene expression patterns in basal-like patients. *n* = 141. (**A**) Associations of TNFα (**A1**), IL-1β (**A2**) and IFNγ (**A3**) with PD-L1. (**B**) Associations of TNFα (**B1**), IL-1β (**B2**) and IFNγ (**B3**) with PD-L2. Correlation coefficients and *p* values are demonstrated in the Figure. Statistical analyses were performed as described in Section 2.

TCGA analyses of basal-like patients (*n* = 141) greatly supported the connection between the pro-inflammatory cytokines and PD-L1/PD-L2 expression (Figure 3). Very significant associations were revealed between high expression levels of TNFα, IL-1β and IFNγ and elevated levels of PD-L1 and PD-L2. As expected in view of the major roles reported for IFNγ in inducing PD-L1 expression, the strongest correlation was between the expression levels of IFNγ and PD-L1 in the patient cohort (r = 0.794, *p* = 1.029 × 10^−31^). IFNγ was also prominently connected to the expression of PD-L2 (r = 0.748, *p* = 1.963 × 10^−26^). Agreeing with the ability of IL-1β to act together with TNFα and IFNγ to promote the extent of TNBC cells co-expressing PD-L1 + PD-L2 (in MDA and BT cells, respectively), IL-1β also demonstrated a relatively high correlation with PD-L1 and PD-L2 expression (with PD-L1: r = 0.604, *p* = 2.348 × 10^−15^; with PD-L2: r = 0.623, *p* = 1.628 × 10^−16^). The association of TNFα with PD-L1 and PD-L2 expression was lower, but nevertheless highly significant (with PD-L1: r = 0.440, *p* = 4.729 × 10^−8^; with PD-L2: r = 0.467, *p* = 5.312 × 10^−9^).

Taken together, the results presented in this part of the study indicate that pro-inflammatory cytokines are important inducers of PD-L1 and PD-L2 expression by TNBC cells, acting together to up-regulate the proportion of cells that co-express the two inhibitory immune-modulating molecules PD-L1 and PD-L2.

### 3.2. Upon IFNγ + IL-1β Stimulation of BT-549 Cells, Signals Are Channeled into STAT1-Mediated Induction of PD-L1 + PD-L2 Co-Expressing Cells

**Figure 4:**
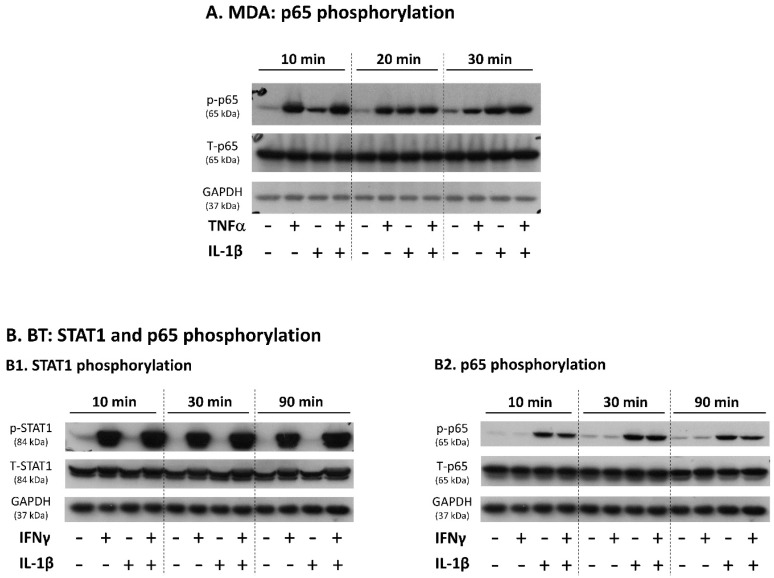
Stimulation of MDA-MB-231 cells by TNFα + IL-1β and of BT-549 cells by IFNγ + IL-1β induces the activation of canonical transcription factors. (**A**) Activation of p65 was determined by kinetics analyses of TNFα and/or IL-1β stimulation in MDA-MB-231 cells (MDA) (cytokine concentrations as in Figure 1). (**B**) Activation of STAT1 (**B1**) and p65 (**B2**) was determined by kinetics analyses of IFNγ and/or IL-1β stimulation in BT-549 cells (BT) (cytokine concentrations as in Figure 2). Control cells were treated by the vehicle of the cytokines. STAT1 and p65 activation were determined by WB analysis. GAPDH was used as loading control. In each panel, a representative experiment of *n* = 3 is presented.

To gain insight into the mechanisms involved in cytokine-induced up-regulation of PD-L1 + PD-L2 co-expressing TNBC cells, we have analyzed the activation of key transcription factors that are involved in mediating cytokine-induced effects. In the case of MDA cells, we stimulated the cells by TNFα + IL-1β—the most prominent combination that elevated the proportion of PD-L1 + PD-L2 co-expressing cells—and determined the activation of p65 (phosphorylation at S536), the NF-κB subunit that plays key roles in mediating TNFα- and IL-1β-induced signals [78,79,80]. Kinetics studies demonstrated rapid activation of p65 upon TNFα stimulation, already at the 10 min time point (Figure 4A); although some decline was noted with time, p65 remained active also after 30 min of TNFα activation. In parallel, IL-1β-induced p65 activation has reached high levels after 20 min of stimulation. At all times, prominent activation of p65 activation was induced by combined stimulation by the two cytokines together (Figure 4A).

In parallel, BT cells were stimulated by IFNγ + IL-1β, the cytokine combination that induced the most effective elevation in PD-L1 + PD-L2 co-expressing cells. Studies using IFNγ or IL-1β stimulation indicated that each cytokine activated its own canonical pathway: STAT1 was activated by IFNγ and p65 by IL-1β (Figure 4(B1,B2)) (STAT1: phosphorylation at Y701; p65: phosphorylation at S536). Strong activation of STAT1 and p65 was noticed when the cells were stimulated by IFNγ + IL-1β together, and it did not decay until 90 min of stimulation (Figure 4(B1,B2)).

**Figure 5:**
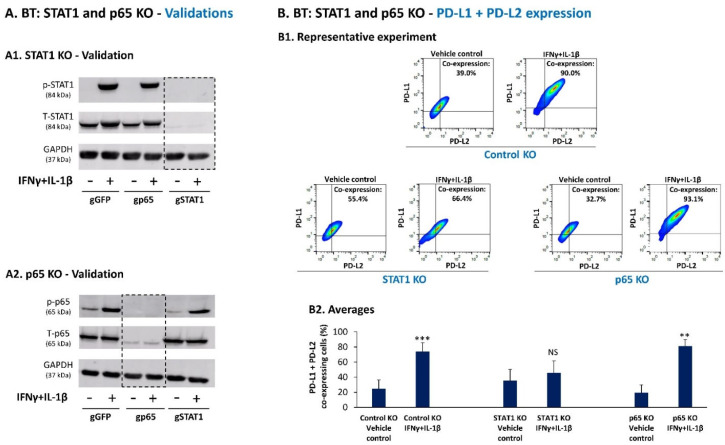
Stimulation of BT-549 cells by IFNγ + IL-1β leads to elevated proportion of PD-L1 + PD-L2 co-expressing cells by activation of STAT1, with no involvement of p65 activation. (**A**) Validation of STAT1 knockout (KO) (**A1**) and p65 KO (**A2**) by CRISPR-Cas9 in BT-549 cells (BT). Activation of STAT1 and of p65 was determined by WB following 30 min stimulation with IFNγ + IL-1β (concentrations as in Figure 2), or in cells treated by the vehicle of cytokines. gGFP, Control KO cells undergoing the CRISPR-Cas9 process with a non-relevant sequence; gSTAT1, Cells in which STAT1 was KO; gp65, Cells in which p65 was KO. In each panel, a representative experiment of *n* = 3 is presented. (**B**) BT cells were stimulated by IFNγ + IL-1β or by the vehicle of the cytokines (conditions as in Figure 2), and the proportion of PD-L1 + PD-L2 co-expressing BT cells was determined by flow cytometry (as described in Figure 1B). (**B1**) A representative experiment of *n* = 3 is presented. (**B2**) Average ± SD of *n* = 3 experiments is presented. *** *p* < 0.001, ** *p* < 0.01. NS, Not significant. Figures S2 and S3 present data on PD-L1 and PD-L2 expression levels (MFI) (single staining) following stimulation by each cytokine alone and together, which are complimentary to data on PD-L1 + PD-L2-co-expresing cells in Figure 5B. Statistical analyses were performed as described in Section 2.

The data of BT cells indicated that STAT1 and p65 were both activated by combined stimulation with IFNγ + IL-1β, and that IL-1β contributed its share to up-regulation of PD-L1 expression levels when it acted in cooperativity with IFNγ. Therefore, we asked if under combined IFNγ + IL-1β stimulation, both transcription factors controlled the expression of PD-L1 and PD-L2 by the cells, or if one of the transcription factors dominated the other. To this end, we generated BT cells in which STAT1 or p65 were knocked out (KO) by CRISPR-Cas9 and were compared to control KO cells (gGFP). Figure 5A demonstrates the results of experiments validating the down-regulation of STAT1 expression and activation in IFNγ + IL-1β-stimulated STAT1 KO cells, and of p65 expression and activation in IFNγ + IL-1β-stimulated p65 KO cells.

Then, flow cytometry analyses were performed in order to determine the impact of STAT1 KO and p65 KO on the ability of IFNγ + IL-1β to promote the percentage of BT cells that co-expressed PD-L1 + PD-L2. As in original BT cells that were not exposed to CRISPR Cas9 manipulation (Figure 2), also in the control KO cells, IFNγ and IL-1β increased cooperatively the expression levels of PD-L1 (MFI) (Figures S2 and S3); also, as in the original cells, combined stimulation of control KO cells by IFNγ + IL-1β has increased the expression of PD-L2 (MFI) (Figures S2 and S3), as well as of the proportion of cells co-expressing PD-L1 + PD-L2 (Figure 5B).

The data of Figure 5B also indicate that in p65 KO cells, combined IFNγ + IL-1β stimulation has increased the proportion of PD-L1 + PD-L2 co-expressing cells, to the same extent identified in control KO cells. In contrast, unlike the response of control KO and p65 KO cells, in STAT1 KO cells the cytokines did not elevate the proportion of PD-L1 + PD-L2 co-expressing cells (Figure 5B). Figures S2 and S3 complement these results by showing the effects of STAT1 KO and p65 KO on the sole expression levels (MFI) of PD-L1 or PD-L2, following stimulation by each of the cytokines alone and together.

These findings indicate that although IL-1β induced p65 activation and acted together with IFNγ to promote PD-L1 expression levels, STAT1 was the actual and sole transcription factor leading to the up-regulation of the proportion of PD-L1 + PD-L2 co-expressing BT cells.

### 3.3. In TNBC Cells, Pro-Inflammatory Cytokines Regulate the Expression of sTNFR1 and sTNFR2 in Divergent Manners

**Figure 6:**
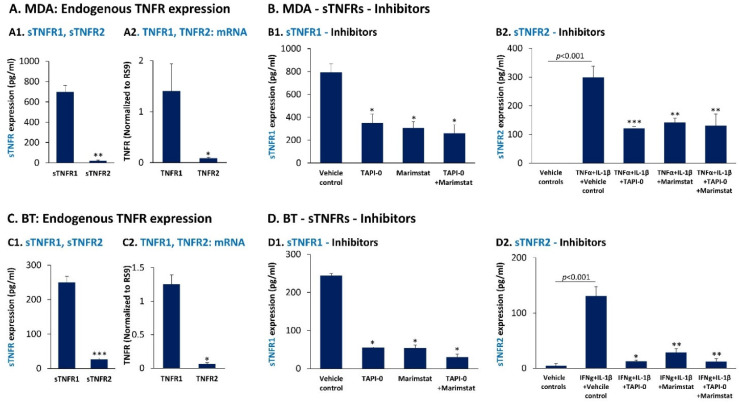
MDA-MB-231 and BT-549 cells express higher levels of cell-derived sTNFR1 than sTNFR2, and both soluble receptors are shed in an ADAM17-dependent process. (**A**,**C**) The constitutive cell-derived expression levels of sTNFR1 and sTNFR2 were determined by ELISA in CM of MDA-MB-231 cells (MDA) (**A1**) and BT-549 cells (BT) (**C1**), after 48 h of cell growth. In parallel, mRNA levels of TNFR1 and TNFR2 were determined by qPCR in MDA cells (**A2**) and BT cells (**C2**), after 5 h of cell growth. In each panel, a representative experiment of *n* = 3 is presented. *** *p* < 0.001, ** *p* < 0.01, * *p* < 0.05. (**B**,**D**) Cell-derived levels of sTNFR1 (**B1**,**D1**) and sTNFR2 (**B2**,**D2**) were determined in a 48 h CM of MDA cells (**B1**,**B2**) and in BT cells (**D1**,**D2**) following treatment with marimastat (3.3 µg/mL), TAPI-0 (5 µg/mL) and both inhibitors together (same concentrations), as indicated. Inhibitor concentrations were selected as described in Section 2 and they did not affect tumor cell growth. To enable determination of the inhibitors on sTNFR2 levels, the experiments in Parts B2 and D2 were performed in the presence of cytokine stimulation: TNFα + IL-1β for MDA cells and IFNγ + IL-1β for BT cells (cytokine concentrations were as in Figure 1 and Figure 2, respectively). Control cells were treated by the vehicle of the inhibitors and/or of the cytokines. In each panel, a representative experiment of *n* = 3 is presented. *** *p* < 0.001, ** *p* < 0.01, * *p* < 0.05. Statistical analyses were performed as described in Section 2.

In view of published studies on the potential roles of sTNFR1 and sTNFR2 as regulators of inflammation [55,56,57,58,59], we now determined the ability of the pro-inflammatory cytokines that regulated PD-L1 and PD-L2 expression to also control the expression levels of sTNFR1 and sTNFR2 in TNBC cells. Prior to studying the impact of the pro-inflammatory cytokines, we analyzed the constitutive cell-derived levels of sTNFR1 and sTNFR2 produced by the TNBC cells included in our study. The findings of Figure 6(A1,C1) demonstrate that the cell-derived protein levels of sTNFR1 were prominently higher than those of sTNFR2 in both MDA and BT cells. This difference in protein levels between sTNFR1 and sTNFR2 was in line with the higher expression levels of TNFR1 than TNFR2 at the mRNA levels, in both cell types (Figure 6(A2,C2)), respectively).

**Figure 7:**
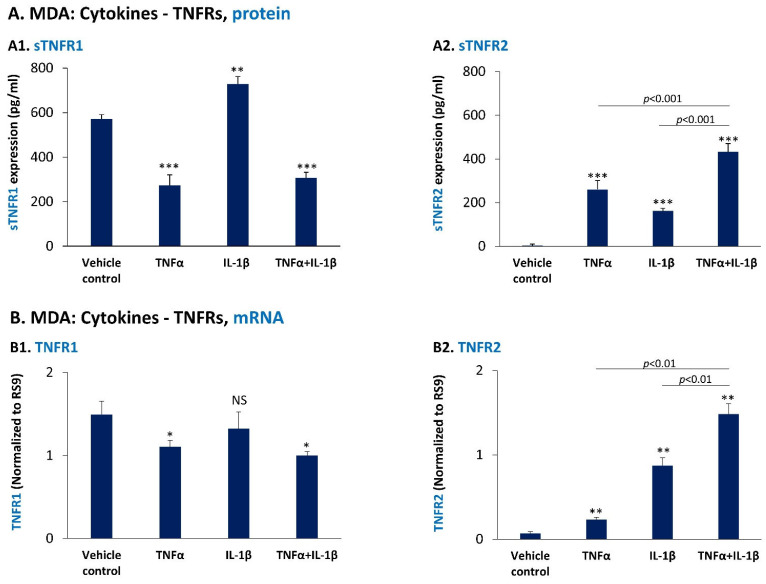
Pro-inflammatory cytokines up-regulate the expression of cell-derived sTNFR2 by MDA-MB-231 cells, but have a divergent effect on cell-derived sTNFR1 expression levels. (**A**) MDA-MB-231 cells (MDA) were stimulated by TNFα and/or IL-1β (concentrations as in Figure 1) or treated by a vehicle control for 48 h. sTNFR1 (**A1**) and sTNFR2 (**A2**) levels were determined in CM of the cells by ELISA. In each panel, a representative experiment of *n* = 3 is presented. *** *p* < 0.001. ** *p* < 0.01. (**B**) MDA cells were stimulated by TNFα and/or IL-1β (concentrations as in Figure 1) or treated by a vehicle control for 5 h. TNFR1 mRNA (**B1**) and TNFR2 mRNA (**B2**) levels were determined by qPCR. In each panel, a representative experiment of *n* = 3 is presented. ** *p* < 0.01, * *p <* 0.05. NS, Not significant. Statistical analyses were performed as described in Section 2.

**Figure 8:**
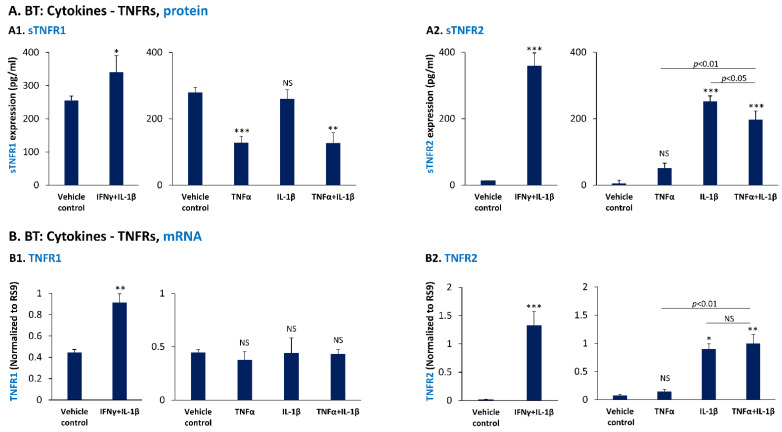
Pro-inflammatory cytokines up-regulate the expression of cell-derived sTNFR2 by BT-549 cells, but have a divergent effect on cell-derived sTNFR1 expression levels. (**A**) BT-549 cells (BT) were stimulated by IFN + IL-1β (concentrations as in Figure 2), by TNFα and/or IL-1β (concentrations as in Figure 1) or treated by a vehicle control for 48 h. sTNFR1 (**A1**) and sTNFR2 (**A2**) levels were determined in CM of the cells by ELISA. In each panel, a representative experiment of *n* = 3 is presented. *** *p* < 0.001, ** *p* < 0.01, * *p* < 0.05. NS, not significant. (**B**) BT cells were stimulated by IFNγ + IL-1β (concentrations as in Figure 2), TNFα and/or IL-1β (concentrations as in Figure 1) or treated by a vehicle control for 5 h. TNFR1 mRNA (**B1**) and TNFR2 mRNA (**B2**) levels were determined by qPCR. In Panel (**B1**), the data of TNFα and/or IL-1β stimulation demonstrate the average ± SD of *n* = 4 experiments, due to high variability between the assays. In all other figures of Panel (**B**), a representative experiment of *n* = 3 is presented. *** *p* < 0.001, ** *p* < 0.01, * *p* < 0.05. NS, not significant. Statistical analyses were performed as described in Section 2.

Previous studies indicated that the soluble forms of TNFα receptors may be derived from alternative splicing or due to cleavage of the membrane receptors by enzymes that belong to the metalloprotease family, with major roles attributed to ADAM17 (A disintegrin and metalloproteinase), known also as TACE (tumor necrosis factor α converting enzyme) [57,59,81,82,83,84]. To determine whether receptor cleavage is involved in the production of cell-derived sTNFR1 and sTNFR2 by TNBC cells, we used the broad spectrum metalloproteinase inhibitor marimastat [85] and the ADAM17 inhibitor TAPI-0 [86] which did not affect tumor cell growth. In view of the fact that cell-derived sTNFR2 (and TNFR2 mRNA) were expressed at low levels by the cells (Figure 6A,C), cytokine stimulation was performed in sTNFR2 studies; this stimulation has led to the production of sTNFR2 by the cells (Figure 7(A2) and Figure 8(A2)), enabling us to determine the impact of the inhibitors on sTNFR2 expression.

When marimastat was used in MDA and BT cells, it has led to a prominent reduction in the levels of cell-derived sTNFR1 and sTNFR2 (Figure 6B,D), indicating that metalloproteases play key roles in generating sTNFR1 and sTNFR2, through a cleavage-mediated process. Moreover, as indicated by the use of TAPI-0 alone or together with marimastat, our findings identified ADAM17 as the major enzyme responsible for cleavage of the membrane receptors, and generating sTNFR1 and sTNFR2 in both TNBC cell types (Figure 6B,D).

Next, we asked whether sTNFR1 and sTNFR2 levels are regulated in TNBC cells by the pro-inflammatory cytokines that have been effective in inducing PD-L1 and PD-L2 expression. When we studied MDA cells, we found out that IL-1β mildly up-regulated the expression levels of sTNFR1 by the cells (Figure 7(A1)); in contrast, TNFα has significantly down-regulated the production of sTNFR1 by the cells and dominated IL-1β-induced effects as demonstrated by reduced sTNFR1 expression following combined TNFα + IL-1β stimulation (Figure 7(A1)); moreover, the mRNA levels of TNFR1 have been down-regulated by TNFα and also by TNFα + IL-1β stimulation (Figure 7(B1)), similar to the effect observed at the protein level of sTNFR1 (Figure 7(A1)). These finding suggest that TNFα down-regulates the transcription levels of TNFR1 and consequently of its shed form, detected at the protein level. In contrast to the effect of TNFα and IL-1β on sTNFR1 levels, each of the two cytokines and more so both of them together, have led to a prominent elevation of sTNFR2 protein levels (Figure 7(A2)), which was also noted at the mRNA levels of TNFR2 (Figure 7(B2)).

Analysis of the impact of IFNγ + IL-1β on sTNFR1 protein expression in BT cells demonstrated a significant elevation (Figure 8(A1)), which corresponded well to IFNγ + IL-1β-induced elevation in TNFR1 mRNA levels (Figure 8(B1)). Furthermore, sTNFR2 levels were up-regulated by IFNγ + IL-1β stimulation, and their effect was noted at the mRNA level of TNFR2 as well (Figure 8(A2,B2)).

In view of the significant roles of TNFα and/or IL-1β in controlling the expression of sTNFR1 and sTNFR2 in MDA cells, we also determined their impact on sTNFR1 and sTNFR2 expression in BT cells. Our data indicate that as in MDA cells, sTNFR1 levels were down-regulated by TNFα—alone and in combination with IL-1β—whereas sTNFR2 was up-regulated by TNFα and/or IL-1β stimulation (Figure 8(A1,A2)). Whereas the protein expression levels of sTNFR1 mRNA (Figure 8(A1)) upon TNFα and/or IL-1β stimulation did not correspond well to the TNFR1 mRNA levels (Figure 8(B1)), protein sTNFR2 levels (Figure 8(A2)) reflected the increase in TNFR2 mRNA levels (Figure 8(B2)), upon cytokine stimulation.

These findings reveal the ability of pro-inflammatory cytokines—at specific settings of stimulation by TNFα, IL-1β and IFNγ alone or in combination—to promote TNFR2 transcription, leading to increased levels of shed sTNFR2 by TNBC cells. In contrast, the expression of sTNFR1 was differently regulated by the pro-inflammatory cytokines: whereas the combination of IFNγ + IL-1β up-regulated the expression of sTNFR1 and TNFR1 mRNA levels in BT cells, TNFα—alone and together with IL-1β—down-regulated sTNFR1 levels in both TNBC cell types; these latter effects on sTNFR1 reflected the regulation of TNFR1 at the mRNA level in MDA cells, but not in BT cells.

Of note, the down-regulation of sTNFR1 levels upon TNFα-containing stimulations was identified as a generalized effect, identified not only in MDA and BT cells, but also in human MDA-MB-468 TNBC cells (Figure S4); similarities between these different TNBC cell lines were also reported in our past research that studied chemokine production and NF-κB/JNK phosphorylation in stroma-TNBC co-cultures [22]. In contrast, the up-regulation of sTNFR2 by the cytokines in MDA and BT cells was not reproduced in MDA-MB-468 cells, indicating that this parameter is prone to different regulatory mechanisms in different TNBC cells, manifesting processes of tumor inter-heterogeneity.

Overall, our findings demonstrate that pro-inflammatory cytokines strongly up-regulate the transcription and consequently the levels of shed sTNFR2, while having divergent regulatory effects on sTNFR1 levels.

### 3.4. sTNFR1 and sTNFR2 Exhibit Potential Anti-Metastatic Functions by Inhibiting TNFα-Induced Production of Inflammatory Pro-Metastatic Chemokines by TNBC Cells

The production of sTNFR1 and sTNFR2 by TNBC cells, and their reported ability to limit inflammatory processes, have led us to question whether the two soluble receptors can modulate the pro-inflammatory and tumor-related characteristics of the cancer cells, which are induced by the ligand of TNFR1 and TNFR2, namely TNFα. To determine this aspect, we have looked specifically at the ability of TNFα to promote in TNBC cells the production of the pro-inflammatory chemokines CXCL8, CXCL1, CCL5 and CCL2, in the presence of recombinant sTNFR1 (rsTNFR1) and rsTNFR2. Of note, these chemokines are identified as powerful inflammatory factors that have potent pro-metastatic activities in cancer [87,88,89,90,91,92,93,94,95,96].

**Figure 9:**
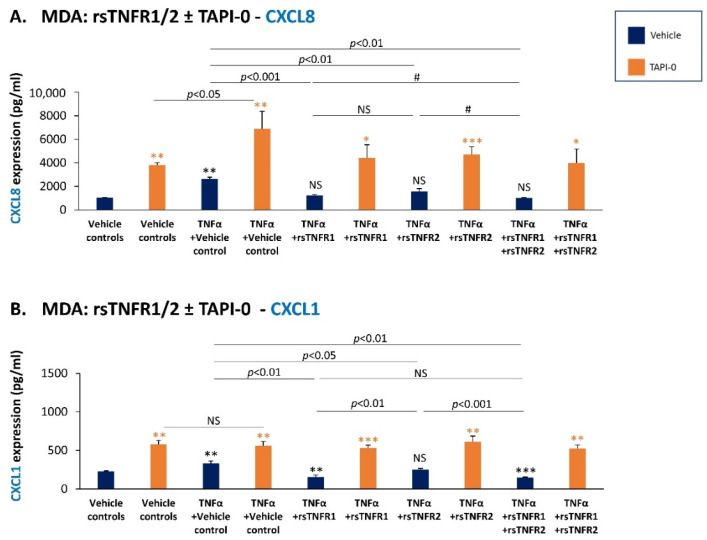
Recombinant and cell-derived sTNFR1 and sTNFR2 have a protective effect against TNFα-induced expression of pro-metastatic chemokines in MDA-MB-231 cells. MDA-MB-231 cells (MDA) were stimulated by TNFα (0.5 ng/mL) that was pre-incubated with rsTNFR1 (150 ng/mL), rsTNFR2 (500 ng/mL), rsTNFR1 + rsTNFR2 (concentrations as before) or their vehicle. When indicated, the cells were cultured prior to TNFα stimulation with TAPI-0 (5 µg/mL) or its vehicle for 3 h, as well as during cytokine stimulation (TAPI-0 did not affect tumor cell growth). The concentrations of rsTNFR1 and rsTNFR2 were selected as described in Section 2. CM were collected following 24 h stimulation, and CXCL8 (**A**) and CXCL1 (**B**) levels were determined by ELISA. A representative experiment of *n* = 3 is presented. *** *p* < 0.001, ** *p* < 0.01, * *p* < 0.05. # *p* < 0.1. NS, not significant. Black asterisks denote the differences in chemokine levels between TNFα-stimulated cells and vehicle-treated cells. Orange asterisks denote the differences in chemokine levels between TAPI-0-treated cells and cells treated by its vehicle. Statistical analyses were performed as described in Section 2.

**Figure 10:**
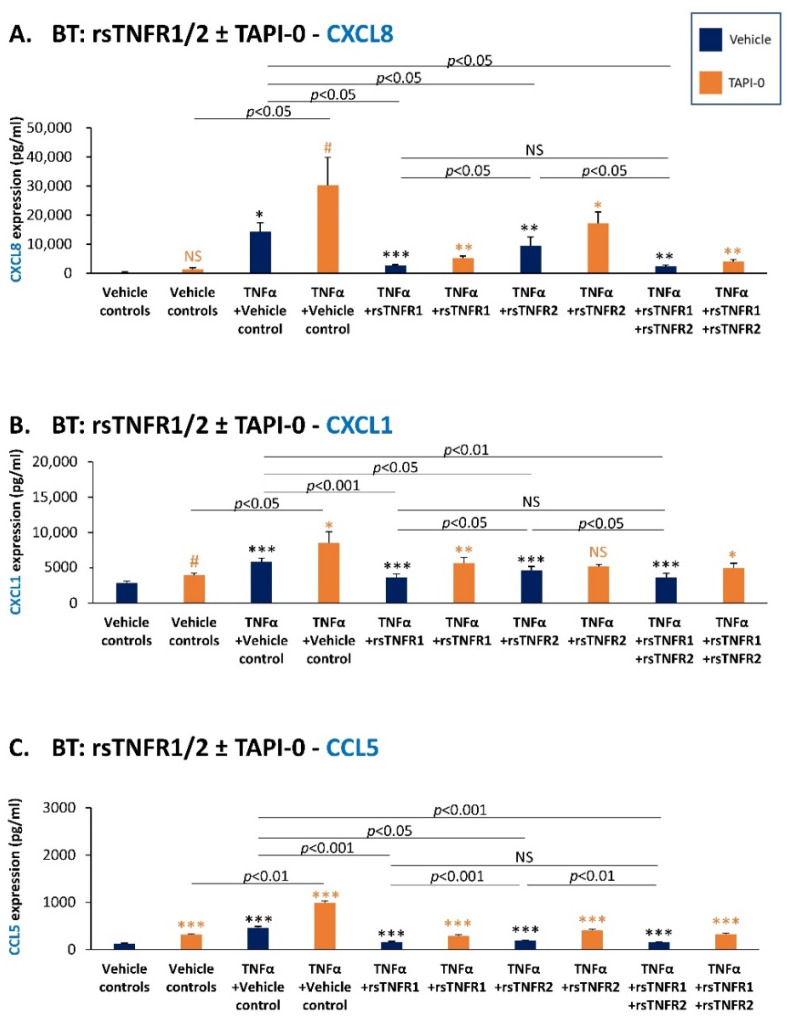
Recombinant and cell-derived sTNFR1 and sTNFR2 have a protective effect against TNFα-induced expression of pro-metastatic chemokines in BT-549 cells. BT-549 cells (BT) were stimulated by TNFα (0.25–0.5 ng/mL) that was pre-incubated with rsTNFR1 (150 ng/mL), rsTNFR2 (500 ng/mL), rsTNFR1 + rsTNFR2 (concentrations as before) or their vehicle. When indicated, the cells were cultured prior to TNFα stimulation with TAPI-0 (5 µg/mL) or its vehicle for 3 h, as well as during cytokine stimulation (TAPI-0 did not affect tumor cell growth). The concentrations of rsTNFR1 and rsTNFR2 were selected as described in Section 2. CM were collected following 24 h stimulation, and CXCL8 (**A**), CXCL1 (**B**) and CCL5 (**C**) levels were determined by ELISA. A representative experiment of *n* = 3 is presented. *** *p* < 0.001, ** *p* < 0.01, * *p* < 0.05, # *p* < 0.1. NS, not significant. Black asterisks denote the differences in chemokine levels between TNFα-stimulated cells and vehicle-treated cells. Orange asterisks denote the differences in chemokine levels between TAPI-0-treated cells and cells treated by its vehicle. Statistical analyses were performed as described in Section 2.

Thus, following dose-dependent analyses that have determined the most effective doses of rsTNFR1 and rsTNFR2, rsTNFR1 and rsTNFR2 were incubated with TNFα prior to its addition to the cells. TNFα that was pre-incubated with the recombinant soluble receptors, or their vehicle control, was then added to the cells, followed by determination of chemokine levels in CM (Figure 9 and Figure 10).

The findings of Figure 9 and Figure 10 demonstrate that in both MDA and BT cells, TNFα that was not pre-incubated with the soluble receptors gave rise to a significant increase in the release of CXCL8 and CXCL1 (dark blue bars; Figure 9A,B and Figure 10A,B), as well as of CCL2 (Figure S5). CCL5 was detected only in BT cells, and was also up-regulated by TNFα stimulation (Figure 10C). When rsTNFR1 and rsTNFR2 were added to TNFα prior to its addition to the cells, they reduced the ability of TNFα to up-regulate the production of CXCL8, CXCL1 and CCL5 (Figure 9 and Figure 10); rsTNFR1 and rsTNFR2 had lower yet significant ability to reduce the production of CCL2 by the cells (Figure S5). These findings indicate that the soluble receptors compete with the membrane receptors on TNFα binding, the result being the lower ability of TNFα to stimulate the cells and lead towards elevated production of the chemokines. Of interest is the fact that rsTNFR2 had, in general, a lower ability to compete with TNFα activities than sTNFR1, despite its use in a higher concentration than rsTNFR1 (rsTNFR1: 150 ng/mL; rsTNFR2: 500 ng/mL).

At this point, we also determined the impact of TAPI-0—the ADAM17 inhibitor that prevented the production of cell-derived sTNFR1 and sTNFR2 (Figure 6B,D)—on TNFα-induced expression of the pro-metastatic chemokines (Figure 9 and Figure 10). The results of these studies (TAPI-0-treated cells are denoted in orange) clearly indicated that in the presence of TAPI-0, the levels of CXCL8, CXCL1 and CCL5 were strongly up-regulated compared to vehicle-treated cells in all treatments, including in the presence of rsTNFR1 and rsTNFR2 (no effect was noted in the case of CCL2; Figure S5).

Taken together with our findings showing that TAPI-0 prevented sTNFR1/sTNFR2 shedding (Figure 6B,D) and the ability of rsTNFR1/rsTNFR2 to inhibit TNFα-induced production of the chemokines (Figure 9 and Figure 10), our data suggest that the TAPI-0-induced elevation in chemokine levels was mediated by its ability to reduce the levels of cell-derived sTNFR1 and sTNFR2. Thus, in the presence of TAPI-0, the down-regulation of cell-derived sTNFR1 and sTNFR2 (and possibly also higher levels of TNFR1/TNFR2 left on the cell membrane) may stand in the basis of the increased ability of TNFα to induce the production of the pro-metastatic chemokines by the cancer cells.

Overall, our findings indicate that sTNFR1 and sTNFR2 inhibit the ability of TNFα-stimulated TNBC cells to release pro-metastatic chemokines that play key roles in tumor progression. Thus, sTNFR1 and sTNFR2 may be considered as protective and anti-malignancy factors in TNBC.

### 3.5. PD-L1 Down-Regulates the Expression of the Anti-Metastatic Factors sTNFR1 and sTNFR2

In view of published studies demonstrating the connection between TNFα/TNFRs and the PD-L1/PD-1 axis [41,42,43,44,45,46] and the high relevance of PD-L1 to TNBC therapy [6,7,8], we have focused on PD-L1 in our further analyses. In this part, we investigated the regulatory interactions that may exist between this inhibitory immune checkpoint and the expression of sTNFR1 and sTNFR2.

**Figure 11:**
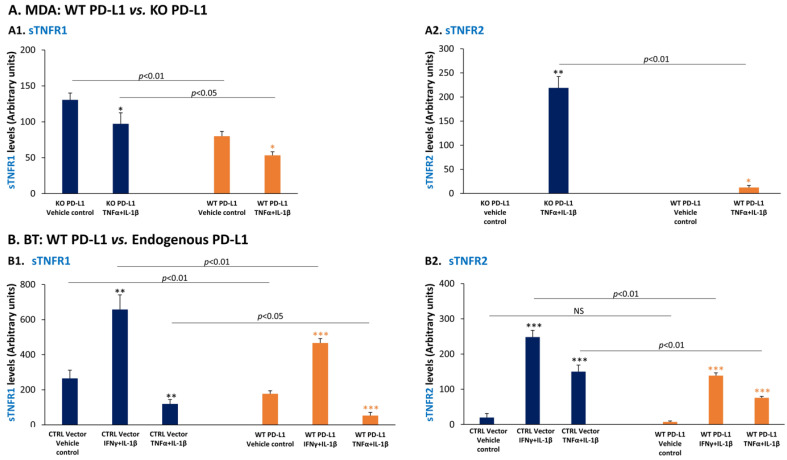
In MDA-MB-231 and BT-549 cells, elevated PD-L1 levels lead to down-regulation of protective sTNFR1 and sTNFR2. (**A**) MDA-MB-231 cells (MDA) and (**B**) BT-549 cells (BT) were treated by pro-inflammatory cytokines or their vehicle control for 48–96 h, and the expression levels of cell-derived sTNFR1 (**A1**,**B1**) and of cell-derived sTNFR2 (**A2**,**B2**) in cell CM were determined by ELISA; sTNFR1 and sTNFR2 levels are presented as arbitrary units, derived from normalization of protein levels to cell numbers, due to higher proliferation rates of WT PD-L1-expressing cells compared to cells that did not express PD-L1 (“KO PD-L1” cells in MDA) or that expressed low endogenous PD-L1 levels (“CTRL Vector” cells in BT). (**A**) MDA cells included cells in which the endogenous expression of PD-L1 was KO by CRISPR-Cas9, and were then infected to express WT PD-L1 (termed “WT PD-L1” cells) or a vector control (“KO PD-L1” cells). Cells of both types were stimulated by TNFα + IL-1β (concentrations as in Figure 1) or treated by vehicle for 48 h. (**A1**) sTNFR1 levels. (**A2**) sTNFR2 levels. The expression levels of PD-L1 by MDA cells have been demonstrated in our published study [66] and are presented again for readers’ convenience in Figure S6A (a different experiment is presented in the current study than in [66]). (**B**) BT cells that expressed endogenous PD-L1 and were infected to over-express WT PD-L1 (“WT PD-L1” cells) were compared to control cells that were infected by a vector control and expressed endogenous, lower, PD-L1 levels (“CTRL Vector” cells). Cells of both types were stimulated by IFNγ + IL-1β (concentrations as in Figure 2) or by TNFα + IL-1β (concentrations as in Figure 1) for 72 h (in Part (**B2**)—for 96 h). Control cells were treated by vehicle. (**B1**) sTNFR1 levels. (**B2**) sTNFR2 levels. The expression levels of PD-L1 by BT cells are shown in Figure S6B (the current BT cells did not express mCherry, unlike the cells in [66]). In all panels, a representative experiment of *n* = 3 is presented. *** *p* < 0.001, ** *p* < 0.01, * *p* < 0.05. NS, not significant. Statistical analyses were performed as described in Section 2.

To this end, we determined the levels of sTNFR1 and sTNFR2 in TNBC cells that expressed high vs. absent/low levels of PD-L1. First, we used MDA cells in which the endogenous expression of PD-L1 was reduced by CRISPR-Cas9, then followed by over-expression of WT PD-L1 (termed “WT PD-L1” cells) or infected by its control empty vector (“KO PD-L1” cells). The expression levels of PD-L1 by the cells were demonstrated in [66] and for readers’ convenience are shown again in Figure S6A (a different experiment is presented in the current study than in [66]). Using these cells, we have determined the expression levels of sTNFR1 and sTNFR2; data are presented as arbitrary units following normalization of protein expression levels to cell numbers, due to higher proliferation rates of WT PD-L1 cells compared to KO PD-L1 cells. The findings of Figure 11(A1,A2) demonstrate that the extracellular levels of cell-derived sTNFR1 and sTNFR2 were significantly lower in WT PD-L1 cells than in KO PD-L1 cells (despite the higher proliferation rates of the former compared to the latter). This finding on sTNFR1 was observed when the cells were stimulated by the pro-inflammatory cytokines TNFα + IL-1β and also without cytokine stimulation.

Similar findings were obtained with BT cells, where we compared cells that expressed endogenous PD-L1 at relatively low levels and were infected by a control vector (“CTRL Vector” cells), and their counterparts that were infected to over-express WT PD-L1 (“WT PD-L1” cells) (Figure S6B shows PD-L1 expression in different cells from those used in our published study [66]; the current ones do not express mCherry). The data of Figure 11(B1,B2) demonstrate that although BT cells that expressed WT PD-L1 had a higher proliferation rate than CTRL Vector cells, the former produced lower levels of sTNFR1 and sTNFR2 than the latter. Thus, elevated WT PD-L1 expression levels have led to the down-regulation of sTNFR1 and sTNFR2 compared to cells that expressed low PD-L1 levels, in the presence of stimulation by IFNγ + IL-1β or by TNFα + IL-1β, as well as in their absence (Figure 11(B1,B2)).

Together, these findings illustrate a regulatory mechanism which is mediated by PD-L1, reducing the expression of sTNFR1 and sTNFR2 that have protective anti-malignancy roles. In view of our published findings indicating that higher expression levels of PD-L1 in TNBC cells lead to increased extracellular levels of the pro-metastatic chemokines CXCL8, CXCL1 and CCL5 [66], our current observations propose that by inhibiting sTNFR1 and sTNFR2 expression, PD-L1 contributes to increased production of the pro-metastatic chemokines, and thus to the pro-malignancy potential of the cancer cells.

## 4. Discussion

Chronic inflammation has been strongly connected to the progression of malignant diseases, by virtue of its ability to promote the pro-metastatic activities of the cancer cells and of the TME [9,10,11,12]. In parallel, recent studies demonstrated that pro-inflammatory processes counteract the ability of ICBs to down-regulate malignancy cascades. Between others, several findings were published on the ability of pro-inflammatory mediators to control the expression of PD-L1 by cancer cells [39,48,49,97,98], but the effects on cancer-related parameters were not explored in depth.

Our current study adds to this research direction by revealing that pro-inflammatory cytokines act in cooperativity to up-regulate the proportion of TNBC cells that co-express the immune inhibitory molecules PD-L1 + PD-L2. Moreover, our findings suggest that PD-L1 can contribute to cancer progression by modes other than suppressing anti-tumor immunity [1,99] and promoting the pro-metastatic functions of tumor cells by cell-autonomous and PD-1-induced manners [66]; this novel pathway may be mediated by PD-L1-induced down-regulation of cell-derived sTNFR1 and sTNFR2—that inhibit the ability of TNBC cells to produce pro-metastatic chemokines such as CXCL8, CXCL1 and CCL5—and therefore are protective and anti-metastatic. Taken together with our published findings showing that increased PD-L1 expression by TNBC cells leads to elevated levels of the exact same pro-metastatic chemokines (CXCL8, CXCL1 and CCL5 [66]), our data suggest that PD-L1-induced down-regulation of cell-derived sTNFR1 and sTNFR2 leads to up-regulation of pro-metastatic chemokines that contribute to chronic inflammation at the tumor site.

Thus, our research proposes protective and anti-malignancy roles for sTNFR1 and sTNFR2 in TNBC. This possibility is supported by a study demonstrating that cell-derived sTNFR2 has reduced the ability of factors derived from murine TNBC 4T1 cells to induce macrophage migration [58]. Taken together with the results of our current study, the findings suggest that sTNFR2 prevents the generation of macrophage-chemoattracting chemokines—such as CCL5—by the cancer cells; in this way, sTNFR2 may reduce the ability of the cancer cells to recruit deleterious tumor-associated macrophages to the tumor site.

Our novel findings provide an important support to the ability of chronic inflammation to potentiate tumor progression, among others, through up-regulation of immune checkpoints. Moreover, they suggest that previous studies that observed high sTNFR1 and/or sTNFR2 levels in breast cancer patients, compared to healthy individuals, as well as investigations showing they were related to poor prognosis and tended to be reduced by chemotherapy [63,64,65], reflect complex interactions that are not fully resolved at this point. The elevated presence of sTNFR1 and sTNFR2 receptors—that are derived mainly from the cleavage of mTNFR1 and mTNFR2 (as we have shown specifically in TNBC cells and others have demonstrated in other systems)—may be a mere reflection of the expression levels of TNFR1 and TNFR2 at the cell membrane, and do not necessarily testify for causative roles of the soluble receptors in promoting disease course. Actually, it is possible that if it was not for sTNFR1 and sTNFR2 exerting protective anti-malignancy effects by TNFα inhibition, disease progression would be more severe.

Other parameters also need to be considered when the connection between breast cancer progression and the expression levels of sTNFR1 and sTNFR2 is considered. First, the studies that have been published so far in breast cancer did not distinguish between the different subtypes of disease, and it is not clear whether the connection of sTNFR1/2 to disease course stands valid in TNBC, when analyzed independently of other breast cancer subtypes. Second, those studies did not determine what the sTNFR1 and sTNFR2 levels were in patient samples, compared to PD-L1 levels and to the levels of TNFα, IL-1β and IFNγ—each alone and together. Such a research direction, which we wish to follow in our future investigations, may shed light on the interactions between these parameters in different breast cancer subtypes.

In the present study, we demonstrated that the pro-inflammatory cytokines also regulate the expression of sTNFR1 and sTNFR2, but in divergent manners. TNFα, the ligand of mTNFR1 and mTNFR2 as well as of sTNFR1 and sTNFR2, acted in negative feedback to down-regulate the expression of sTNFR1 in several TNBC cell types (MDA-MB-231, BT-549, MDA-MB-468). Here, it is important to note that the levels of sTNFR1 produced in TNBC cells were relatively high (Figure 6(A1,C1)), and thus they may strongly interfere with the ability of TNFα to induce the expression of pro-metastatic chemokines, as shown in Figure 9 and Figure 10. Under these conditions, it is in the “interest” of TNFα—as a pro-tumor factor—to down-regulate the expression of protective sTNFR1, in that way, leading to higher levels of pro-metastatic chemokines at the TME.

In this context, it is interesting to note that TNFα had an opposite effect on sTNFR2 expression, leading to its up-regulation in TNBC cells. sTNFR2 elevation was also noted upon stimulation by the other pro-inflammatory cytokines (IL-1β, IFNγ) and their combinations (TNFα + IL-1β in the case of MDA cells and BT cells and IFNγ + IL-1β in BT cells). The reason for this opposing effect on sTNFR2—compared to sTNFR1—may be in the fact that sTNFR2 is released by TNBC cells in relatively low levels; and more so, that it may have a smaller ability to counteract TNFα activities, compared to sTNFR1. This difference between the two soluble receptors is proposed by the fact that rsTNFR2 was generally less effective than rsTNFR1 in inhibiting the ability of TNFα to induce the expression of the pro-metastatic chemokines, and had to be used in a higher concentration (Figure 9 and Figure 10).

Moreover, sTNFR2 may possibly have much more complex activities in the tumor milieu than initially expected. In parallel to acting as an anti-malignancy factor through competition with mTNFR2 on the binding of TNFα (a case in which the “interest” of the tumor cells is to down-regulate sTNFR2 expression under conditions of elevated PD-L1 expression), sTNFR2 may induce reverse signaling by its preferred ligand, which is transmembrane TNFα (tmTNFα) [100,101,102]. Such processes of tmTNFα-activated pathways have led to NF-κB activation and to pro-tumor effects in cancer cells, thus serving their needs at yet another level [101,102]. Under such conditions, stimulation of the cancer cells by pro-inflammatory cytokines may favor the elevation of sTNFR2 levels, as they may increase disease progression. Thus, equilibrium between two pathways—PD-L1-induced down-regulation of sTNFR2 and inflammation-induced up-regulation of sTNFR2 levels—may eventually dictate the equilibrium between the protective and tumor-promoting activities of TNFR2 in TNBC.

## 5. Conclusions

Our present study has provided novel evidence for the roles of an inflammation-driven network in inducing pro-metastatic activities of TNBC cells, by up-regulating the expression of the inhibitory immune checkpoints PD-L1 and PD-L2, and controlling the levels of soluble TNFα receptors. Moreover, we have shown that elevated expression of PD-L1 has down-regulated the protective anti-tumor factors sTNFR1 and sTNFR2, and consequently may lead to elevated expression of pro-metastatic chemokines.

sTNFR1 and sTNFR2 were found in previous studies to compete on TNFα binding with mTNFR1 and mTNFR2, and have anti-inflammatory effects [55,56,57,58,59]. In our current research, the two soluble receptors inhibited the ability of TNFα to stimulate the production of inflammatory chemokines by TNBC cells. Hence, it is possible that sTNFR1 and sTNFR2 have the capacity to reduce the potency of pro-inflammatory signals that are mediated by TNFα in TNBC tumors, and thus limit the level of chronic inflammation in tumors. The ability of PD-L1 to counteract the protective functions of sTNFR1 and sTNFR2 suggests that the efficacy of ICBs directed to the PD-L1/PD-1 pathway may increase by combining them with the inhibition of TNFα activities, or of its membrane receptors.

Along these lines, several recent studies that were performed in mouse murine systems indicated that such a combination-based approach could indeed be advantageous in inhibiting cancer progression. For example, TNFα/TNFR1 deficiency, as well as antibodies to TNFα, have improved the ability of anti-PD-1 antibodies to reduce tumor load and increase survival in a melanoma model [41]. Other investigations demonstrated the ability of TNFR2 inhibitory measures to improve the anti-tumor effects of ICBs directed to PD-L1/PD-1 [42,43,44].

Thus, our findings suggest that the combined administration of ICBs with inhibitors of TNFα/TNFRs is a path that needs to be considered in TNBC therapy. This mode of treatment is achievable, because of the routine use of inhibitors of TNFα activities in autoimmune and inflammatory diseases [103,104]. Along these lines, a 2021 clinical trial has estimated the safety and efficacy of combined treatment by anti-PD-1 + anti-CTLA-4 + certolizumab/infliximab that target TNFα [105]. This study demonstrated the safety of these treatment modes; partial or complete responses were noted in all patients treated by the certolizumab-containing treatment, and in about half of the patients who were given the infliximab-containing treatment [105].

Taken together, our findings point at complex inflammation-driven interactions in TNBC, regulating the expression of PD-L1/PD-L2 and sTNFR1/sTNFR2, as well as the impact of the soluble TNFα receptors on the pro-metastatic functions of TNBC cells. The clinical implications of this research, together with other studies in the field, emphasize the need to consider combined treatments that target the TNFα/TNFRs and PD-L1/PD-1 pathways together, in TNBC therapy.

## Data Availability

Data supporting reported results presented in this study are available on request from the corresponding author.

## Supplementary Materials

The following supporting information can be downloaded at: https://www.mdpi.com/article/10.3390/cancers14143513/s1, Figure S1. The effect of TNFα + IL-1β stimulation on the proportion of PD-L1 + PD-L2 co-expressing BT-549 cells; Figure S2. The impact of IFNγ and/or IL-1β on PD-L1 or PD-L2 expression in BT-549 cells in which STAT1 was knocked out; Figure S3. The impact of IFNγ and/or IL-1β on PD-L1 or PD-L2 expression in BT-549 cells in which p65 was knocked out; Figure S4. The impact of TNFα and/or IL-1β on sTNFR1 expression in MDA-MB-468 cells; Figure S5. The impact of rsTNFR1/2 and/or of TAPI-0 on the release of CCL2 by MDA and BT cells; Figure S6. PD-L1 expression by the cells used in Figure 11. File S1. Full western blot images.
