# Supplementary material for: Inflammation-Driven Regulation of PD-L1 and PD-L2, and Their Cross-Interactions with Protective Soluble TNFα Receptors in Human Triple-Negative Breast Cancer
Source: Cancers (Basel). 2022 Jul 19;14(14):3513. doi: 10.3390/cancers14143513 (PMC9323351; doi:10.3390/cancers14143513)
Supplement: Supplementary file 1 [file cancers-14-03513-s001.zip › Ben-Baruch-Supplementary Figures-Final-15.7.22.pdf]

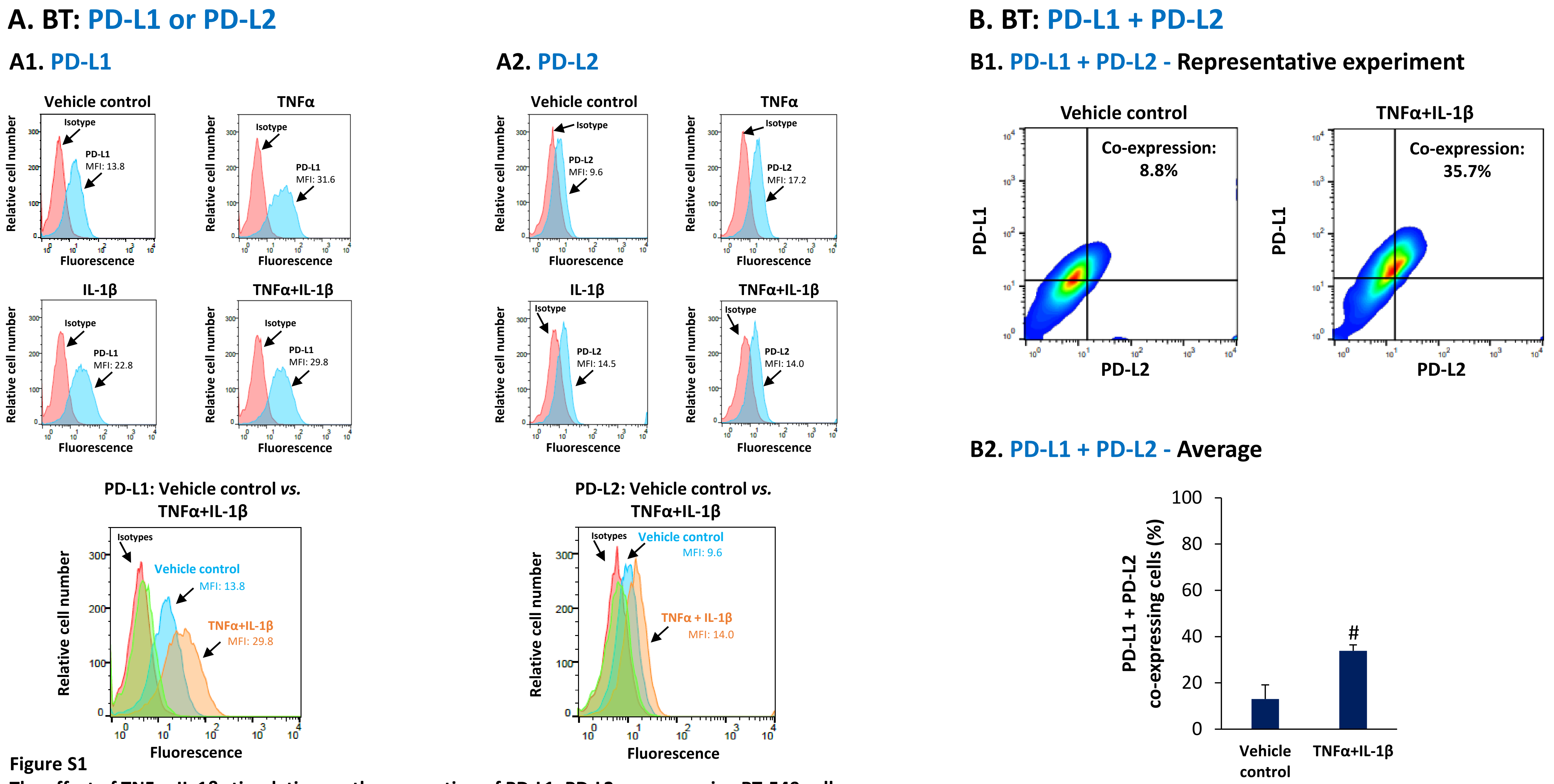

Figure S1

The effect of TNFα+IL-1β stimulation on the proportion of PD-L1+PD-L2-co-expressing BT-549 cells

(**A**) BT-549 cells (BT) were stimulated by TNFα and/or IL-β (TNFα: 50ng/ml; IL-1β: 500pg/ml) for 24 hours. Control cells were treated by the vehicle of the cytokines. Cytokine concentrations were selected as described in Materials and methods. Cell surface expression of PD-L1 (A1) and PD-L2 (A2) was determined by flow cytometry; MFI, Mean fluorescence. Isotype/s, Non-relevant antibodies used as control/s. A representative experiment of n=2 is presented. (**B**) BT cells were stimulated by TNFα+IL-1β or vehicle, as in Part A. The proportion of cells co-expressing PD-L1+PD-L2 was determined by flow cytometry, as described in Figure 1B. (**B1**) A representative experiment of n=2 is presented. (**B2**) Average ± SD of n=2 is presented. #$p < 0.1$. Statistical analyses were performed as described in Materials and Methods.

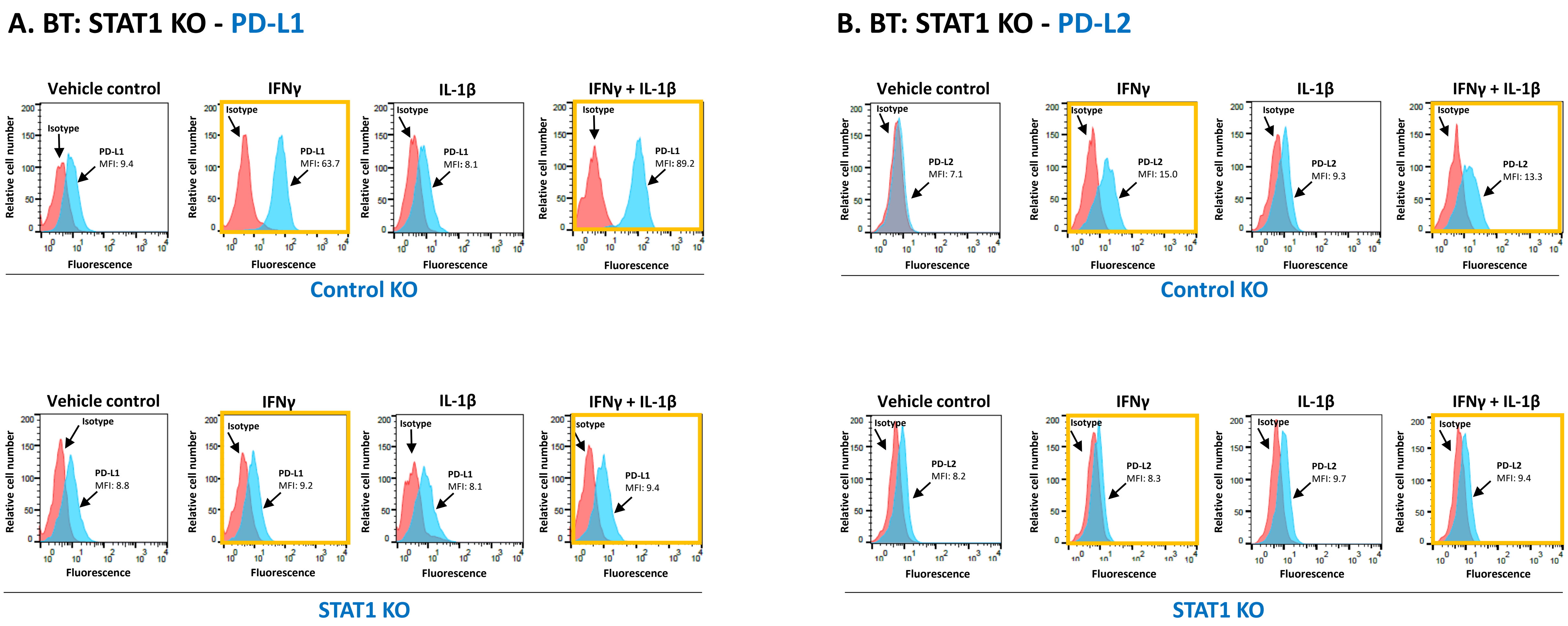

Figure S2

The impact of IFNγ and/or IL-1β on PD-L1 or PD-L2 expression in BT-549 cells in which STAT1 was knocked-out

The expression of STAT1 was knocked-out (KO) in BT-549 cells (BT). Down-regulation of STAT1 expression and activation was validated as demonstrated in Figure 5A. "Control KO", Cells that were infected with gGFP, used as control. "STAT1 KO", Cells that were infected with gSTAT1. The cells were stimulated by IFNγ and/or IL-β (IFNγ: 20ng/ml; IL-1β: 500pg/ml) for 24 hours. Vehicle control cells were treated by the vehicle of the cytokines. Cell surface expression of PD-L1 (**A**) or of PD-L2 (**B**) was determined by flow cytometry; MFI, Mean fluorescence. Isotype, Non-relevant antibody used as control. A representative experiment of n=3 is presented. Yellow frames highlight cytokine stimulations in which STAT1 down-regulation has reduced the expression of PD-L1 and PD-L2 expression (MFI); Please note that Figure 5, which complements the current Supplementary Figure, demonstrates the impact of STAT1 KO on the proportions of PD-L1+PD-L2-co-expressing cells and not on MFI.

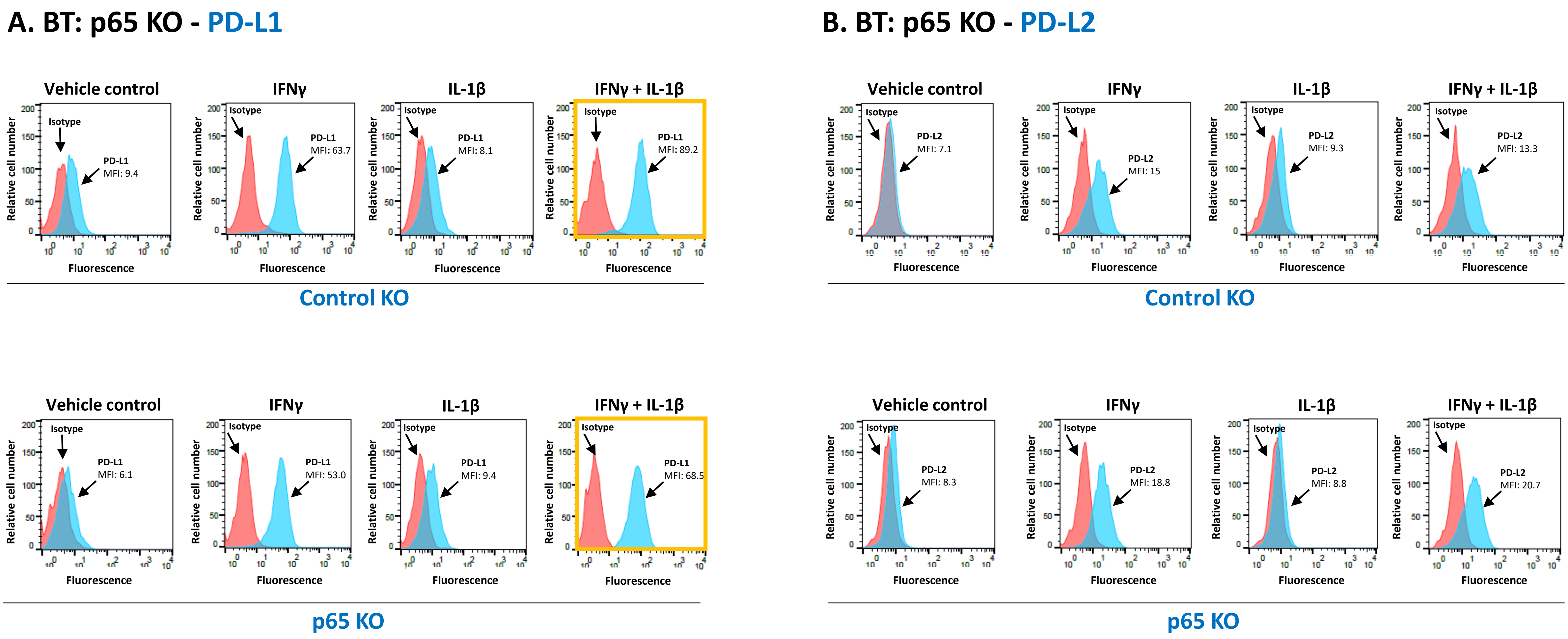

Figure S3

The impact of IFNγ and/or IL-1β on PD-L1 or PD-L2 expression in BT-549 cells in which p65 was knocked-out

The expression of p65 was knocked-out (KO) in BT-549 cells (BT); Down-regulation of p65 expression and activation was validated as demonstrated in Figure 5A. “Control KO”, Cells that were infected with gGFP, used as control. “p65 KO”, Cells that were infected with gp65. the cells were stimulated by IFNγ and/or IL-β (IFNγ: 20ng/ml; IL-1β: 500pg/ml) for 24 hours. Vehicle control cells were treated by the vehicle of the cytokines. Cell surface expression of PD-L1 (**A**) or of PD-L2 (**B**) was determined by flow cytometry; MFI, Mean fluorescence. Isotype, Non-relevant antibody used as control. A representative experiment of n=3 is presented. Yellow frames highlight cytokine stimulations in which p65 down-regulation has reduced the expression of PD-L1+D-L2 expression (MFI); Please note that Figure 5, which complements the current Supplementary Figure, demonstrates the impact of p65 KO on the proportions of PD-L1+PD-L2-co-expressing cells and not on MFI.

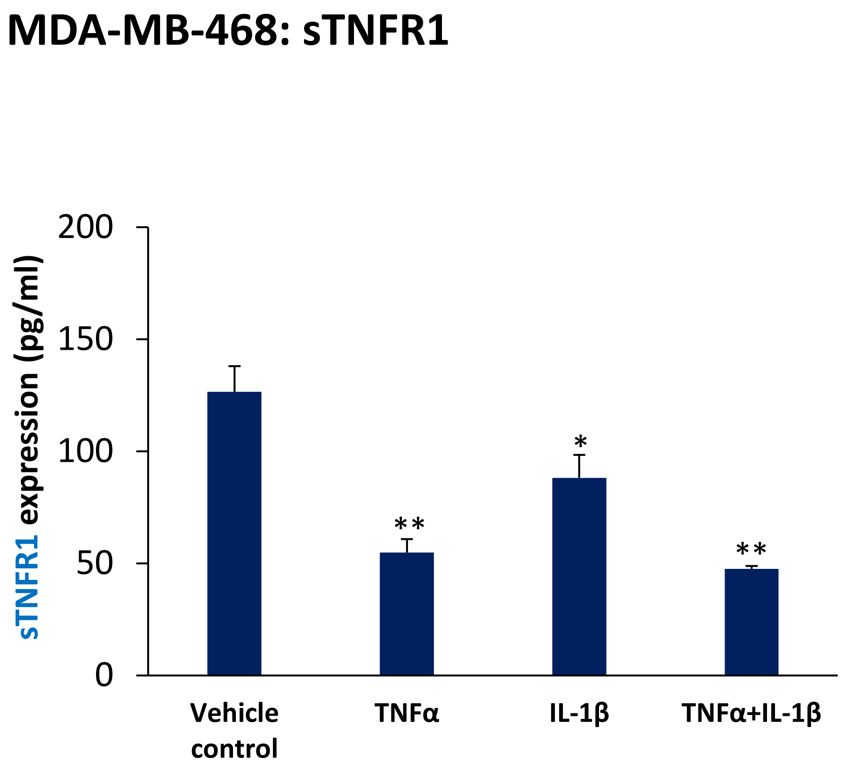

Figure S4

The impact of TNFα and/or IL-1β on sTNFR1 expression in MDA-MB-468 cells

MDA-MB-468 cells were stimulated by TNFα and/or IL-1β (concentrations as in Fig. 1) or treated by a vehicle control for 48 hours. The levels of endogenous sTNFR1 were determined in CM of the cells by ELISA. A representative experiment of n=3 is presented. The expression of endogenous sTNFR2 was not detected in the cells, prior or after cytokine stimulation (data not shown). **$p < 0.01$, *$p < 0.05$. Statistical analyses were performed as described in Materials and Methods.

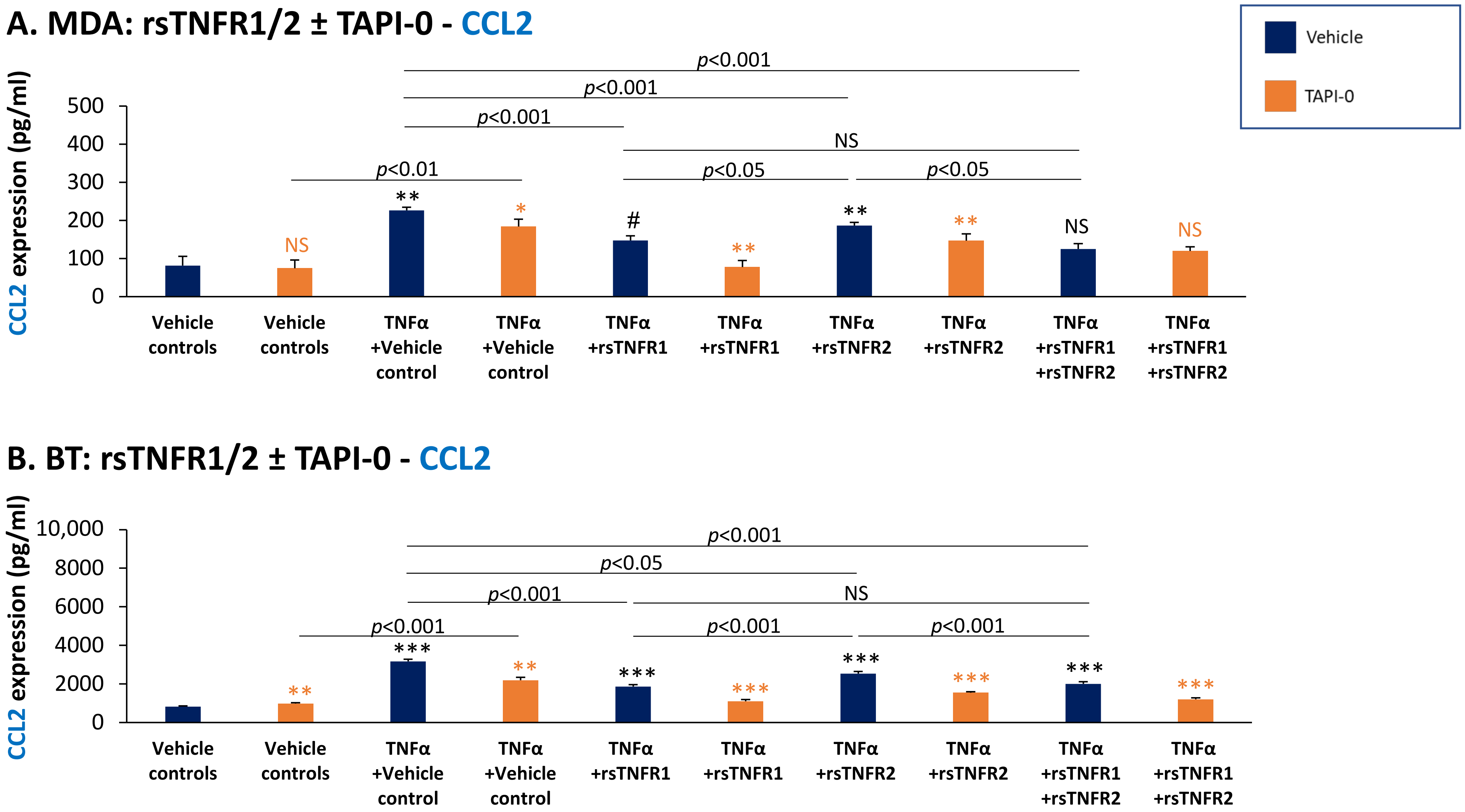

Figure S5

The impact of rsTNFR1/2 and/or of TAPI-0 on the release of CCL2 by MDA-MB-231 and BT-549 cells

MDA-MB-231 cells (MDA) (**A**) and BT-549 cells (BT) (**B**) were stimulated by TNFα (0.5ng/ml) that has been pre-incubated with rsTNFR1 (150ng/ml), rsTNFR2 (500ng/ml), rsTNFR1+rsTNFR2 or their vehicle control. When indicated, the cells were cultured prior to TNFα stimulation with TAPI-0 (5µg/ml) or its vehicle for 3 hours, as well as during cytokine stimulation. The concentrations of rsTNFR2 and rsTNFR1 were selected as described in Material and methods. Cell supernatants were collected and CCL2 levels were determined by ELISA. In each panel, a representative of n=3 experiments is presented. ***$p < 0.001$, **$p < 0.01$, *$p < 0.05$. #p<0.1. NS, Not significant. Black asterisks denote the differences in chemokine levels between TNFα-stimulated cells and vehicle-treated cells. Orange asterisks denote the differences in chemokine levels between TAPI-0-treated cells and cells treated by its vehicle. Statistical analyses were performed as described in Materials and Methods.

A. MDA: PD-L1 expression

B. BT: PD-L1 expression

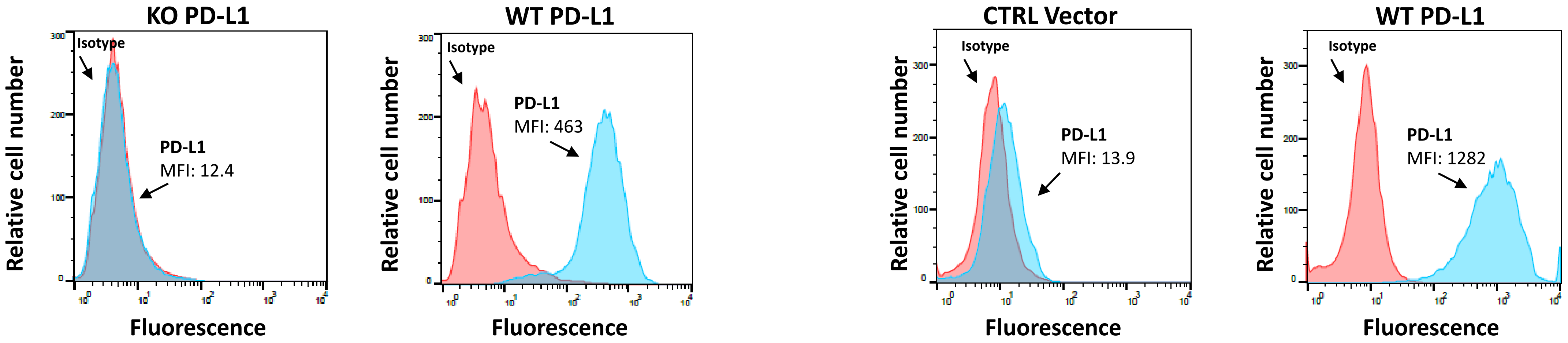

Figure S6

PD-L1 expression by the cells used in Figure 11

(**A**) MDA-MB-231 cells (MDA) included cells in which the endogenous expression of PD-L1 was knocked-out (KO) by CRISPR-Cas9, and were then infected to express WT PD-L1 (termed "WT PD-L1" cells) or with a control vector ("KO PD-L1" cells). (**B**) BT cells included cells that expressed endogenous PD-L1 and were infected to over-express WT PD-L1 ("WT PD-L1" cells); control cells were infected by vector control ("CTRL Vector" cells). The expression of PD-L1 was determined by flow cytometry. MFI, Mean fluorescence intensity. Isotype, a control of an isotype-matched non-relevant antibody.

The expression levels of PD-L1 by MDA cells has been demonstrated in our published study [66] and are presented again for readers' convenience (a different experiment is presented in the current Figure compared to [66]). The expression levels of PD-L1 by BT cells were determined in a new set of cells, which did not express mCherry (unlike the cells used in [66], expressing mCherry).
