# Supplementary figures and images for: Inflammation-Driven Regulation of PD-L1 and PD-L2, and Their Cross-Interactions with Protective Soluble TNFα Receptors in Human Triple-Negative Breast Cancer
Source: Cancers (Basel). 2022 Jul 19;14(14):3513. doi: 10.3390/cancers14143513 (PMC9323351; doi:10.3390/cancers14143513)

Figure 4A

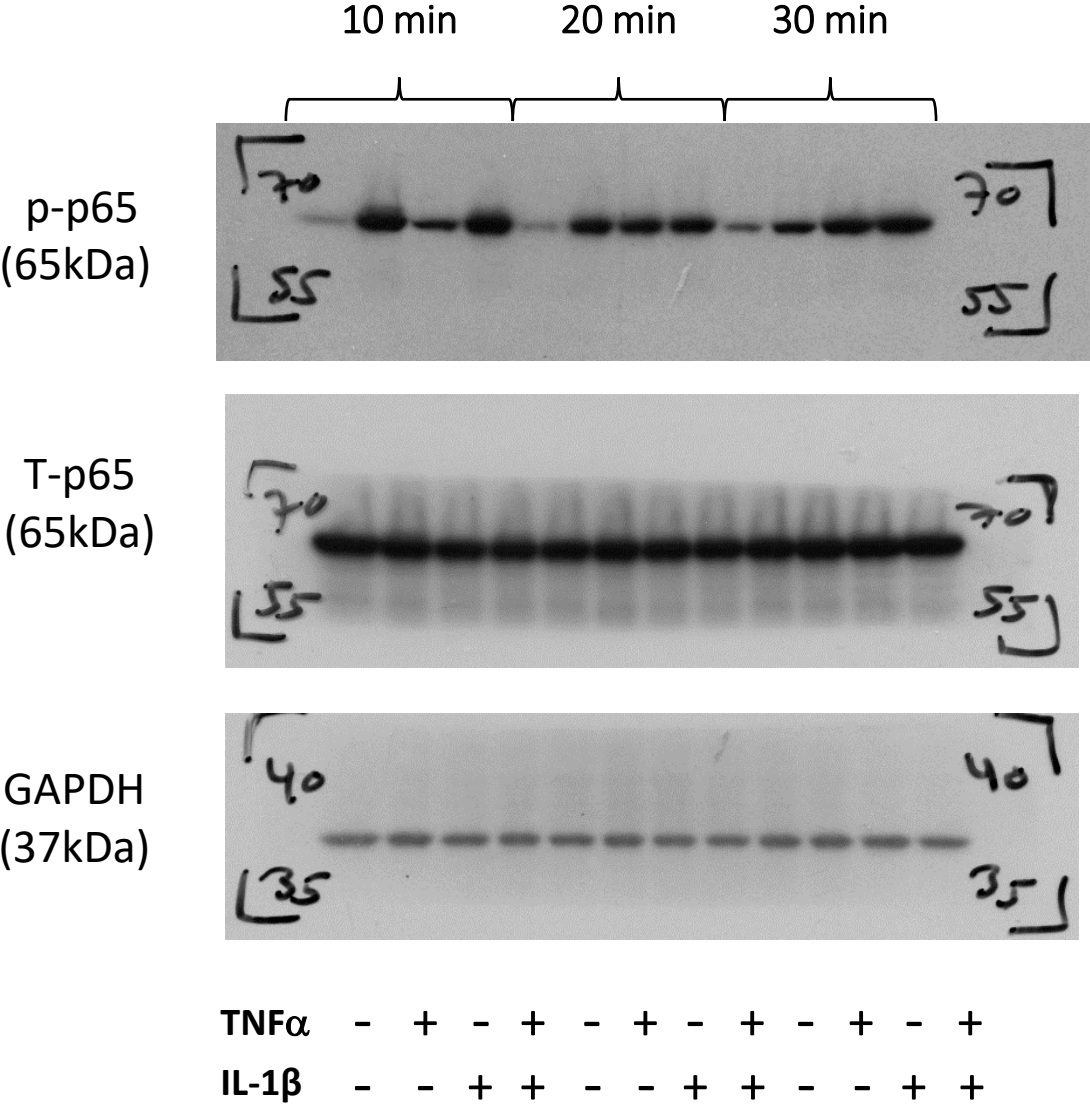

Figure 4B1

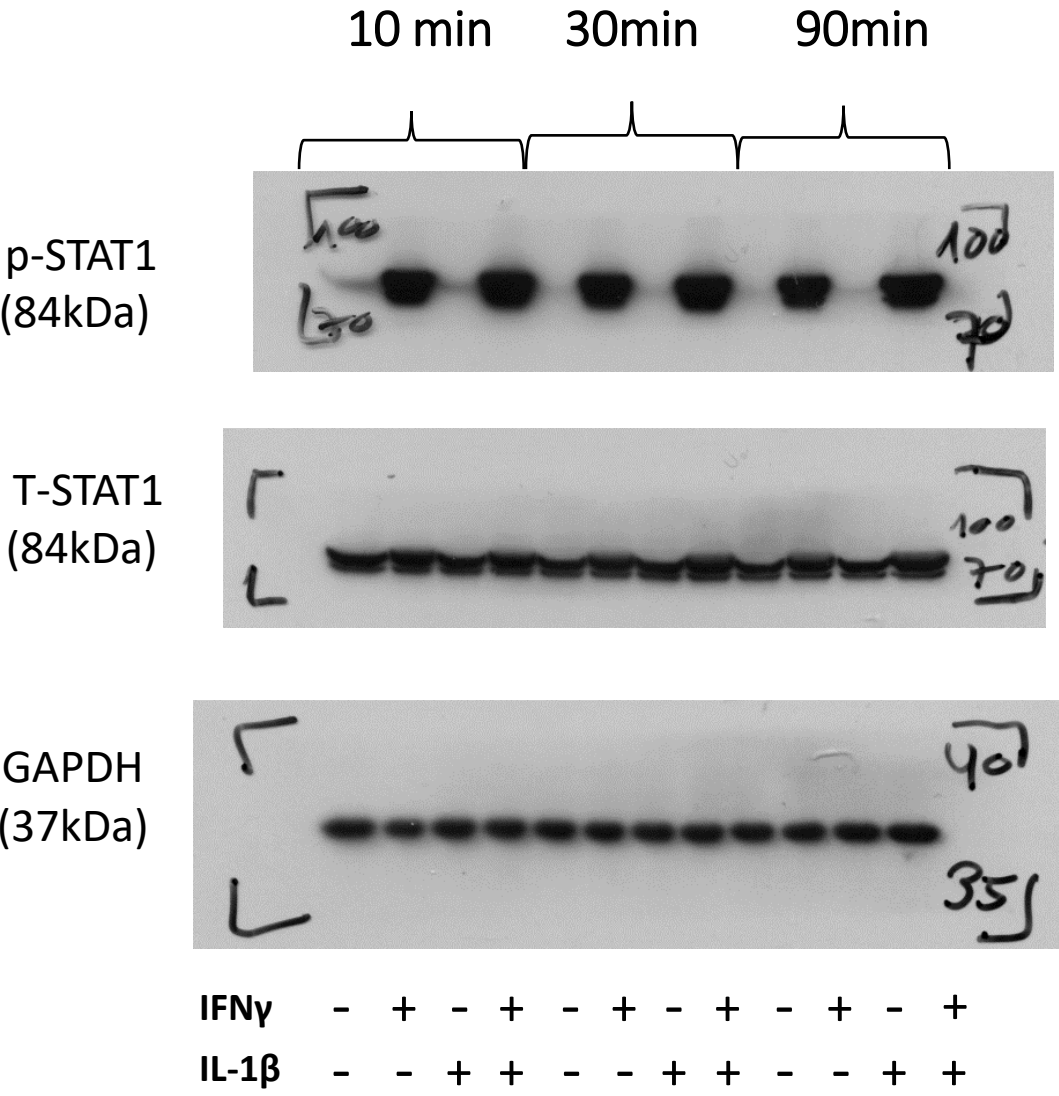

Figure 4B2

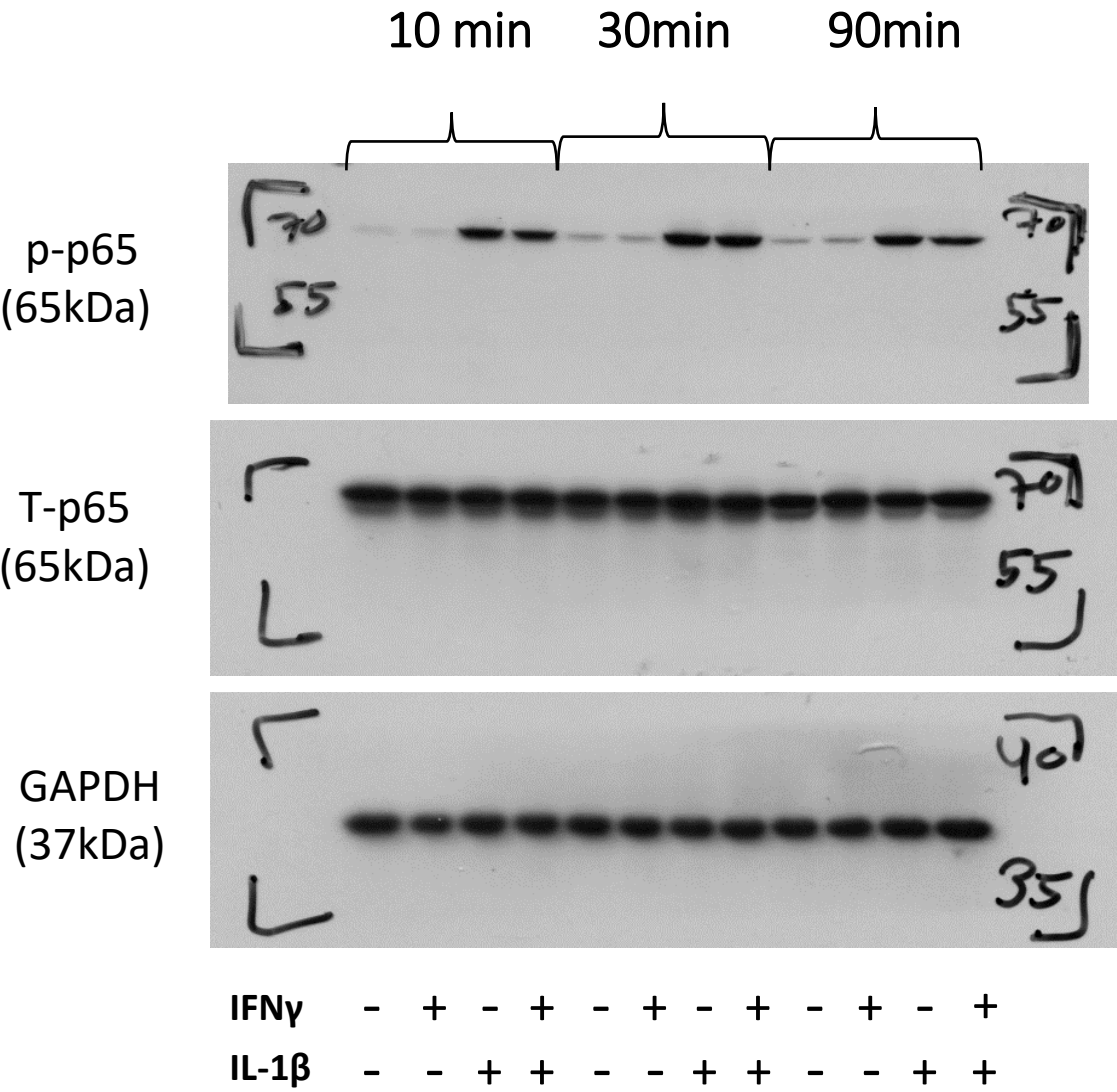

Figure 5A1

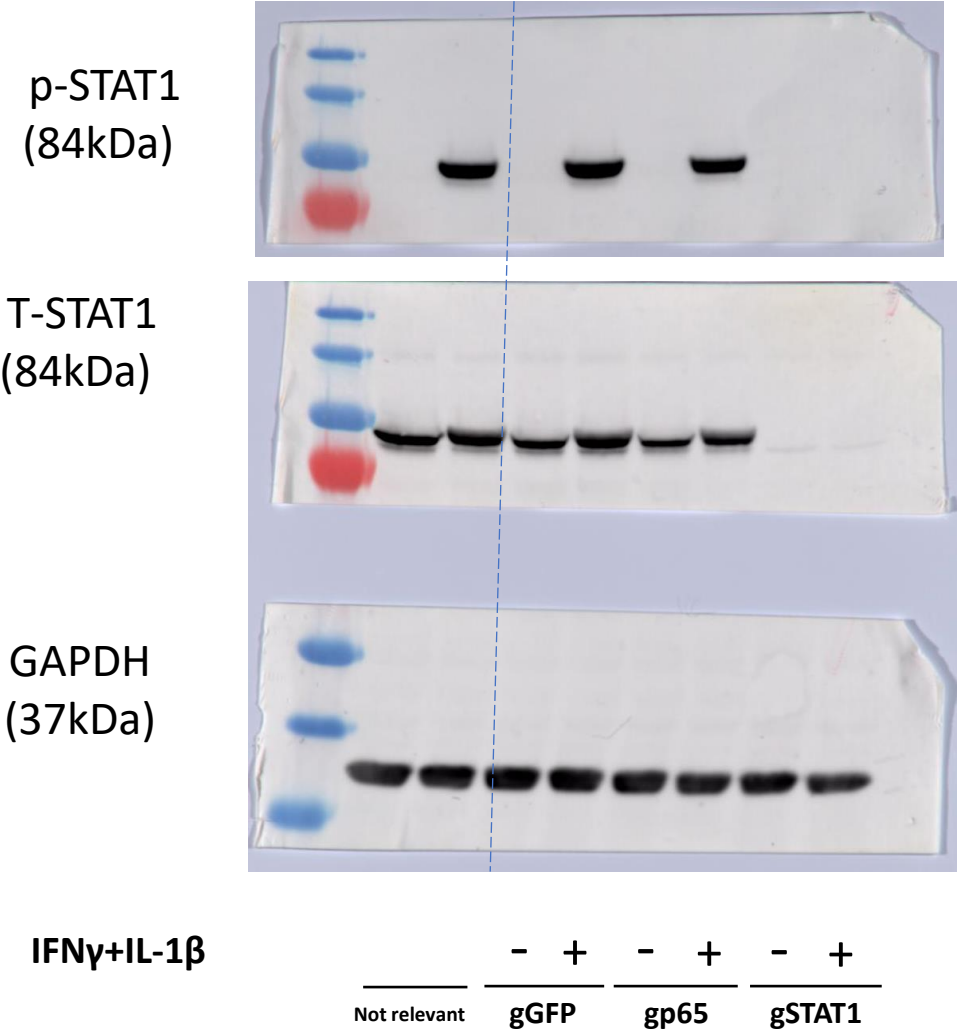

Figure 5A2

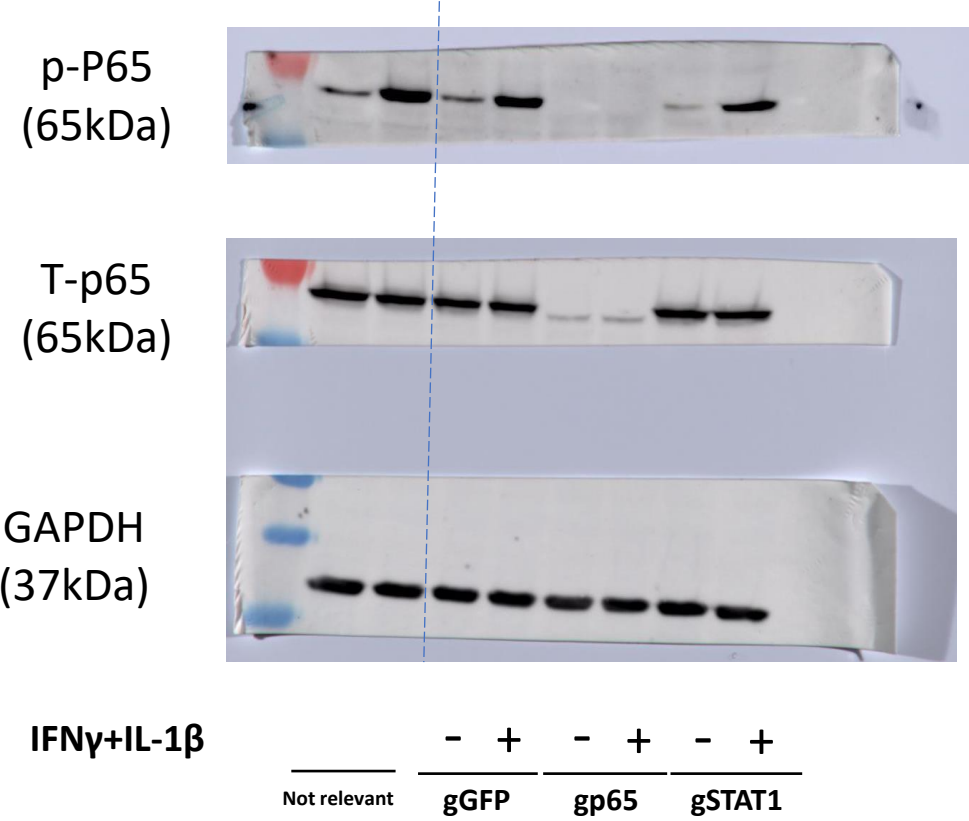

Supplement: Supplementary file 1 [file cancers-14-03513-s001.zip › File S1. Western blot images.pdf]
